# A Chemical Framework for Engineering Extracellular Vesicles’ Biointerface to Advance Precision Therapeutics

**DOI:** 10.1002/adma.74203

**Published:** 2026-07-25

**Authors:** Leila Pourtalebi Jahromi, Felix Geng, Jan‐Noël Frenzke, Gregor Fuhrmann

**Affiliations:** ^1^ Faculty of Science, Department of Biology, Pharmaceutical Biology Friedrich‐Alexander‐Universität Erlangen‐Nürnberg Erlangen Germany; ^2^ FAU NeW Friedrich‐Alexander‐Universität Erlangen‐Nürnberg Erlangen Germany

**Keywords:** bioengineering, bioorthogonal chemistry, chemical biology, extracellular vesicles, surface engineering

## Abstract

Extracellular vesicles (EVs) are membrane‐bound nanoparticles ubiquitously secreted by all cell types and serve diverse physiological and pathological functions. Due to their pivotal roles in pathophysiological processes and their inherent biomimetic properties, EVs have attracted significant attention as biomarkers, as well as for tissue engineering and drug delivery. The surface chemistry of EVs dictates their interactions with their environment. Great strides have been made to tailor this biochemical interface with the aim of enhancing cargo delivery, target specificity, immune evasion, and tracking capabilities, while preserving EVs’ stability and functional integrity. Approaches to surface modification primarily encompass genetic and metabolic manipulation of parent cells, application of physical forces, and chemical reactions. In this manuscript, we introduce a comprehensive chemistry‐centric framework for EV surface engineering that integrates demonstrated EV modification strategies with protein‐ and cell‐surface chemistries not yet applied to EVs, delineating their functional scope and translational potential for advancing EV‐based therapeutics.

## Graphical abstract

Extracellular vesicles (EVs) are cell‐derived nanoparticles that mediate intercellular communication through their dynamic biointerfaces. Owing to their intrinsic biological functions, EVs have emerged as promising platforms for biomedical applications. This review highlights chemical engineering strategies to tailor EV interfaces, enabling enhanced targeting, drug delivery, diagnostics, and controlled interactions with biological systems.

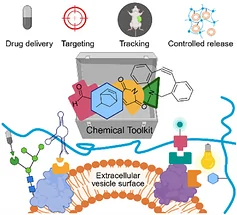

## 1 Introduction

The medical field is rapidly shifting from a “one‐size‐fits‐all” approach toward the paradigm of precision and personalized medicine, in which therapeutic strategies are tailored to the biological characteristics of individual patients, diseases, and target tissues to enhance efficacy while minimizing off‐target effects. Advances in nanotechnology, biotechnology, and modern chemical engineering have driven the emergence of precision nanotherapeutics—engineered nanoscale systems designed to overcome biological barriers through precise control of their size, shape, surface chemistry, responsiveness, and targeting capabilities [1]. In this framework, naturally derived nanoparticles such as patient‐derived extracellular vesicles (EVs) have attracted growing attention, and considerable research efforts are now focused on developing engineering strategies to optimize EVs for biomedical applications.

EVs are membrane‐bound nanoparticles ubiquitously secreted by cells across all domains of life, serving diverse biological purposes. Their molecular composition is remarkably versatile, encompassing small and large biomolecules such as secondary metabolites, lipids, carbohydrates, proteins, and nucleic acids [2]. Despite significant progress, the mechanisms governing the selective sorting of cargo into EVs across various cell types, from prokaryotes to human cells, remain incompletely understood [3]. Over the past five decades, the biogenesis pathways, biological functions, and physiological/pathological roles of EVs have been extensively studied. However, their potential for biomedical applications has attracted significant attention only within the last two decades and in the paradigm of precision medicine [4, 5].

EVs are promising candidates as disease biomarkers, therapeutic agents [6], drug delivery platforms [7], tools for modulating cellular phenotypes [8], and tissue engineering [9] in both research and clinical contexts. These versatile applications are underpinned by EVs’ nanoscale dimensions, their compartmentalized core‐shell architecture enabling the encapsulation and transport of both hydrophobic and hydrophilic molecules, and their biomimetic properties [10].

**FIGURE 1:**
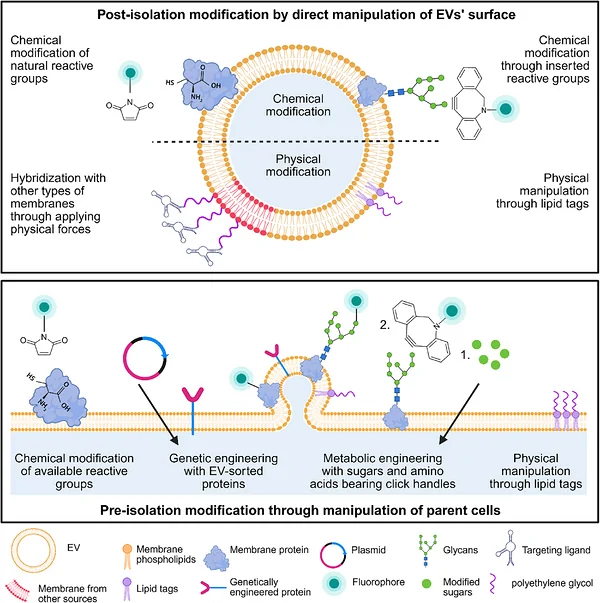
Pre‐ and post‐isolation strategies for surface modification of EVs.

The physicochemical properties of EVs—including their shape, size, surface characteristics, and mechanical attributes—play a pivotal role in determining their stability, biodistribution, cellular interactions, and functionality [11]. Among these, surface parameters such as charge, roughness, and topology, all governed by surface chemistry, have emerged as critical determinants of EVs' biological fate and functionality [12]. Consequently, the surface engineering of EVs to optimize their pharmacokinetic and pharmacodynamic profiles has become a rapidly expanding area of investigation [13]. Figure 1 shows various strategies for EV surface modification, including pre‐ and post‐isolation approaches. Pre‐isolation methods refer to techniques for manipulating the parent cells, including genetic and metabolic engineering, application of physical forces, and chemical reactions, to equip the membrane of produced EVs with therapeutic cargos, fluorescent proteins, and targeting ligands [14, 15]. In contrast, post‐isolation approaches involve directly modifying the surface of EVs rather than engineering their parent cells. These methods include introducing functionalities through physical interactions, hybridizing EVs with other membrane types, and applying chemical engineering techniques.

In this manuscript, we systematically evaluate chemical strategies for EV surface engineering—from conventional conjugation methods to emerging bioorthogonal reactions—to identify practical opportunities and constraints, guiding their development as clinically translatable, personalized nanotherapeutic platforms. These chemical strategies are classified based on their reactivity toward the available functional groups on the surface of EVs (Box 1). Conventional reactions are often scalable and widely applicable, but their limited biocompatibility, environmental sustainability, efficiency, and selectivity—along with harsh operating conditions incompatible with the biological environment—restrict their biomedical applications [16]. In contrast, the Nobel Laureate Karl Barry Sharpless coined the term “click chemistry” to describe modular reactions with a wide scope, high yield, and specificity, proceeding under mild conditions, and bearing products with simple purification, while avoiding offensive byproducts [17]. Another Nobel Laureate, Carolyn R. Bertozzi, took this concept further and used the term “bioorthogonal” to describe reactions that happen within a living system without interfering with natural bioprocesses [18]. This typically requires the introduction of functional groups, naturally absent from biological systems through metabolic engineering (such as introduction of azide‐bearing sugars), harnessing physical interactions (such as phospholipid anchors with azide‐bearing heads), hybridization with synthetic vesicles bearing a specific functional group (such as hybridization of EVs with liposomes made of azide‐modified lipids), or using a bifunctional linker that reacts with native chemical groups from one side and bears a click handle on the other side. In this manuscript, we go beyond established EV surface engineering approaches to emphasize underexplored reactions adapted from biomolecular and cell‐surface engineering.

**Classification of chemical avenues for EV surface engineering**

**Amine‐directed reactions**

1. Schiff base reaction with aldehydes
2. Amide coupling with carboxylic acids and esters
3. Protein‐protein ligation through amine‐directed reactions
4. Thiourea bond formation with isothiocyanates

**Thiol‐directed reactions**

1. Activated alkenes
2. Intein‐mediated protein‐protein ligation
3. Native chemical ligation between thiols and thioesters
4. Other thiol‐reactive functional groups

**Hydroxyl‐directed reactions**

**Carboxylic acid‐directed reactions**

**Ligand‐directed reactions**

**Carbonyl‐directed reactions**

**Reactions directed to reactive carbons of aromatic amino acids**

**Reactions requiring installation of unnatural functional groups**

1. Staudinger ligation of azides and phosphines
2. Azide‐alkyne 1,3‐dipolar cycloaddition
3. Strain‐promoted alkyne‐nitrone cycloaddition
4. Tetrazine ligation
5. Isocyanide reactions
6. Photoclick reactions

**Radiolabeling chemistry**

## 2 Amine‐Directed Reactions

**FIGURE 2:**
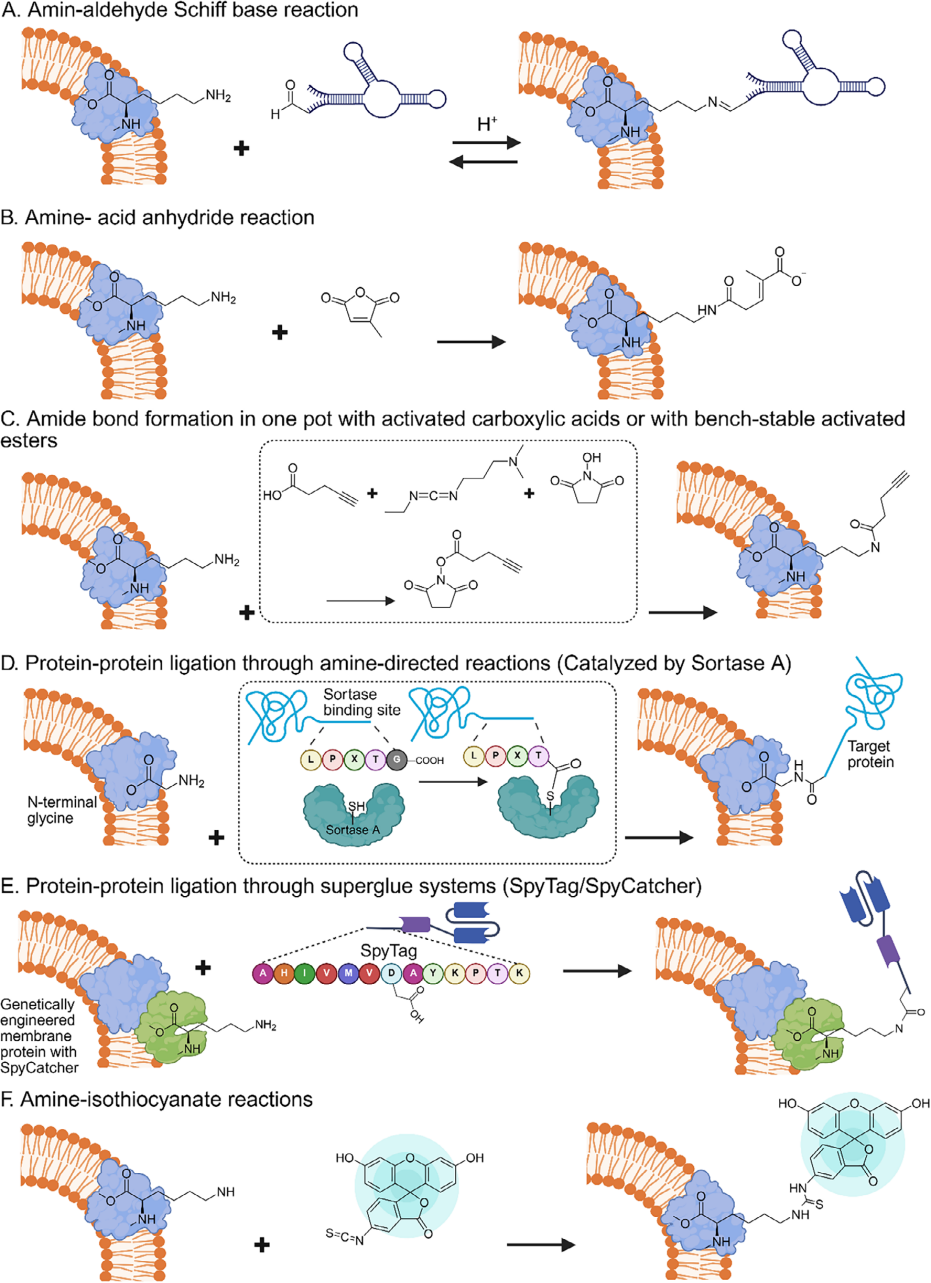
Biocompatible strategies for EVs’ surface modification through N‐terminal amines or lysine side chains.

Lysine's side chain and the N‐terminal of proteins carry a primary amine (−NH_2_), which acts as a diverse platform for nucleophilic reactions. Some reactions show a higher specificity toward amine on the lysine's side chain, because of surface accessibility, relative abundance, and most importantly, a higher pKa and nucleophilicity compared to the N‐terminal amine. Proteins, glycoproteins, peptidoglycans, and lipoproteins on the EVs’ surface can all be addressed through amine‐directed reactions, depicted in Figure 2.

### 2.1 Schiff Base Reaction With Aldehydes

The Schiff base reaction between amines and electron‐poor carbonyl groups has been extensively used in biomaterial engineering to prepare bioadhesive and self‐healing hydrogels [19]. Schiff base bonds (imines) are inherently unstable in aqueous and physiological environments, especially at neutral or acidic pH, where they can hydrolyze back into the initial functional groups; therefore, their formation reaction does not fulfill the criteria of the click reactions. Accordingly, strategies based on the Schiff base reaction are mostly harnessed for the immobilization of EVs within scaffolds and on implants to provide a controlled EV release rather than EV surface decoration [20, 21]. Besides, these strategies are used in emerging biosensor technologies for detecting, quantifying, and characterizing EVs [22]. Nevertheless, there are a few attempts reported in the literature using the Schiff base reaction principle for surface decoration of EVs with specific ligands. For instance, a bone marrow mesenchymal stem cell (BMSC)‐specific aptamer was designed with an aldehyde group at the 5’ terminal to enable targeting BMSC‐derived EVs to bone marrow tissue for promoting bone regeneration in osteoporosis and fractures (Figure 2A) [23]. Visualizing the accumulation of aptamer‐conjugated EVs in animal experiments showed significant retention of the nanoparticles at the bone site for at least 12 h, which implied a relative stability of the imine bond between the aptamer and the EV surface proteins. In future studies, the reversibility of the Schiff base reaction in acidic environments could be considered as an opportunity for immobilizing prodrug or drug molecules via the imine bond on the surface of EVs, enabling on‐demand release in acidic extracellular conditions, including tumor and infection sites, as well as within the cells’ endosomal compartment.

### 2.2 Amide Coupling With Carboxylic Acids and Esters

The conventional reaction between carboxylic acids and esters with amines has been limitedly employed for bioconjugation in mild physiological conditions. The reason is the low efficiency of this reaction due to the equilibrium between carboxylic acid and its deprotonated form, which reduces the electrophilicity of the carbonyl group. The reaction of amines with regular carboxylic acids usually requires catalysts, coupling reagents, or high‐energy input to form reactive intermediates.

Compared to regular carboxylic acids and esters, the activated species’ carbonyl groups are usually more electrophilic and carry better leaving groups, which improves the kinetics of the reaction with an amine, resulting in amide bond formation in milder conditions. Overall, the reactivity of the carbonyl group with amine can be described in the following order: acid chlorides > acid anhydrides > activated esters; nevertheless, the kinetics of specific reactions can be compared only with knowledge about the electrophilicity of the carbonyl carbon dictated by the electron‐demanding atoms/groups on the carbonyl, steric effects, and the inherent stability of the leaving groups.

Acid chlorides [24] and acid anhydrides [25] have long been used for protein synthesis and modification. Yet, their application for EV surface engineering has been limited due to their disadvantages. Acid chlorides are extremely reactive with water and produce HCl as a byproduct, which makes them unappealing for reactions in physiological conditions. Acid anhydrides are more stable than acid chlorides, but still less stable compared to many activated esters, designed for nucleophilic reactions in mild physiological environments.

The reaction between amines and anhydrides has been suggested as a strategy for changing the zeta potential of EVs without significantly affecting their cellular uptake. Researchers have successfully reduced the surface charge of serum‐derived EVs from −15 to −50 mV using the reaction between the EV surface amines with citraconic anhydride (Figure 2B) [26]. This strategy may promote the development of nanocomposites based on electrostatic reactions of highly negative EVs with positively‐charged biopolymers for tissue engineering and drug delivery. The anhydride‐amine reaction has also been used for direct conjugation of bifunctional radioisotope chelators, including diethylenetriaminepentaacetic dianhydride (DTPA‐anhydride) for carrying ^111^In on the EV surface to enable biodistribution studies with single‐photon emission computed tomography (SPECT) [27]. Another example of using anhydrides for the modification of EV's surface proteins is introduced by Wan and colleagues [28]. They utilized the reaction between the amines from the EVs with methyl acrylic anhydride, resulting in the installation of an olefin bond on the EVs’ surface. Further, the amine groups from a targeting peptide were installed on the EVs’ surface through an acylation reaction. This study represents a modular avenue for the on‐demand coupling of amine‐bearing molecules to the surface of EVs.

As mentioned above, a prominent strategy to enable conventional carboxylic acids to react with amines is to use a coupling reagent. The one‐pot reaction of amine groups from EV surface proteins with carboxylic acids in the presence of 1‐ethyl‐3‐(3‐dimethylaminopropyl) carbodiimide (EDC) and N‐hydroxysuccinimide (NHS) is one of the frequently reported methods of addressing amines in bioconjugation literature. In this reaction, EDC activates carboxyl groups by forming an O‐acylisourea intermediate, which is very reactive and prone to hydrolysis in the absence of NHS. However, in the presence of NHS, it forms an NHS ester, a more stable intermediate, highly reactive to amines. Although EDC/NHS chemistry has been employed in protein engineering since 1982 [29], there was a significant time gap until it was first used in 2014 for EV surface modification. Smyth and colleagues first leveraged this principle for coupling surface amines with 4‐pentynoic acid (Figure 2C), whose alkyne group further reacts with an azide‐bearing dye with a copper‐catalyzed azide‐alkyne cycloaddition (CuAAC) mechanism [30]. In further attempts, the EDC/NHS one‐pot reaction was used to install not only dyes but a variety of other functional groups and ligands, including small‐molecule drugs [31], cell‐penetrating peptides [32], tumor‐targeting peptides [33], monoclonal antibodies [34], and aptamers [35] to the surface of EVs.

Despite simplicity, the reaction of EV amines with carboxylic acid‐bearing ligands in the presence of EDC/NHS has some limitations that reduce the yield of the reaction and destabilize protein structure. Specifically, in more acidic conditions, where the aspartates and glutamates’ carboxylic acid side chains are protonated, they can compete with the ligand's carboxylic acid for reacting with EDC. Therefore, using a bench‐stable NHS ester derivative instead of the carboxylic acid derivative of the ligand is more common for EV surface modification.

An early attempt to use NHS esters for surface modification of EVs was introduced in 1989 by Sehested et al. for decorating tumor‐derived plasma membrane vesicles with a Doxorubicin's NHS ester derivative for drug delivery to Doxorubicin‐resistant tumor cells [36]. Since then, NHS ester derivatives of many fluorescent and near‐infrared (NIR) dyes have been introduced in the literature and have been commercialized to enable EV labeling for uptake and biodistribution studies [37, 38]. In addition to dyes, NHS esters have become a standard solution for pegylation [39], installing targeting moieties, including aptamers [40] and proteins. Finally, yet importantly, the NHS esters have been employed for conjugating many bifunctional chemical linkers, including biotin [41], azides [39], and alkynes [42, 43] to the surface of EVs as a platform for further affinity and chemical interactions.

A major drawback of NHS ester derivatives of some ligands compared to the original form is reduced water solubility. To address this problem, a sulfonate group can be added to the NHS ring to produce N‐hydroxysulfosuccinimide ester (sulfo‐NHS ester). This negatively charged group normally increases the solubility of the ligand and makes it less prone to hydrolysis without interfering with the mechanism of the reaction [44]. Another advantage of sulfo‐NHS esters over NHS esters for surface modification of EVs is that, due to a negative charge, the sulfo‐NHS esters do not permeate the EV membrane [29] and direct the reaction exclusively to the surface amines. Accordingly, sulfo‐NHS esters have been reported in the literature for introducing the same categories of chemical modalities introduced to the EVs’ surface with NHS esters, including biotinylation [45, 46] and bifunctional click chemistry linkers [47, 48, 49, 50].

Besides NHS and sulfo‐NHS esters, other classes of activated esters that have been previously used in protein synthesis and engineering can be used for surface modification of EVs. Developed in 1999 [51], 4‐Sulfo‐2,3,5,6‐tetrafluorophenyl (STP) esters have been recently introduced as a more efficient alternative to NHS esters for installing a click chemistry handle, methyl tetrazine, on the surface of EVs [52]. Yet, many other classes of amine‐reactive activated esters, offering increased reactivity and reduced possibility of producing byproducts, including pentafluorophenyl (PFP) esters and 1‐Hydroxybenzotriazole (HOBt) esters, remain to be explored in the context of EV surface modification.

### 2.3 Protein‐Protein Ligation Through Amine‐Directed Reactions

Protein ligation techniques based on mechanisms evolved in nature have been repurposed as chemical biology tools for protein, biomaterial, and cell surface engineering. Here, we describe enzymatic ligation and molecular superglue systems that fall in the amine‐directed reactions category, as such bioinspired mechanisms [53].

Enzymatic ligation is a bioconjugation strategy that employs enzymes to catalyze the formation of covalent bonds between biomolecules, usually through an amine‐directed reaction. Sortase A from *Staphylococcus aureus* and protein ligases, such as asparaginyl endopeptidase from *Oldenlandia affinis* (OaAEP1), are among the enzymes that have been used for the conjugation of specific proteins or peptide‐tagged entities on EVs’ surface. The mechanism behind sortase‐mediated enzymatic ligation is the reaction between amine and thioester (Figure 2D) [54]. Sortase A recognizes and cleaves the bond between the threonine and glycine in a specific recognition motif ‐typically LPXTG, where X can be any amino acid‐ on a substrate protein, leading to the formation of a reactive thioacyl intermediate through a cysteine residue in its active site. Then, the enzyme‐bound thioacyl intermediate is attacked by the nucleophilic amino group from another protein, resulting in peptide bond formation and release of sortase A. This enzymatic reaction has been used for engineering cells [55], biomaterials [56], and nanoparticles [57], as well as surface functionalization of EVs. Erythrocyte EVs with membrane proteins that act as nucleophiles have been previously decorated with epidermal growth factor receptor (EGFR) targeting peptides with a sortase‐specific domain [58]. Also, Glycine‐rich domains can be introduced to the surface of other types of EVs through physical post‐isolation methods to provide a platform for sortase‐mediated conjugation to other proteins and peptides. This strategy has recently been reported for the functionalization of milk‐derived EVs with fluorescent proteins and targeting nanobodies [59]. OaAEP1 is another enzyme used for the installation of targeting peptides and antibodies on the surface of EVs [60, 61], even with faster kinetics and higher efficiency compared to sortase A [58]. OaAEP1 specifically recognizes a peptide sequence motif, usually asparagine‐glycine‐leucine, and cleaves the peptide bond between the asparagine and glycine. Then, the enzyme's active site cysteine (or aspartate) is covalently linked to the carbonyl carbon of the cleaved peptide, followed by the nucleophilic attack of the amine from another peptide [62]. These successful examples of enzymatic protein ligation for EV bioengineering imply the potential for expanding the research field by investigating a variety of ligase enzymes recognizing versatile domains for advancing site‐specific EV surface modification.

Molecular superglue systems are a class of protein ligation tools that achieve irreversible covalent bonding between two interacting proteins or peptides with extremely high specificity and speed [53]. Inspired by isopeptide bond formation in *S. pyogenes* fibronectin‐binding protein, these methods rely on spontaneous bonding between the carboxylate of aspartate (or glutamate) and the amine of lysine in two proteins: a 13‐amino acid SpyTag and a 138‐amino acid SpyCatcher (Figure 2E) [63]. Genetic engineering of cells to display SpyCatcher on EV surface proteins enables modular attachment of SpyTag‐linked targeting peptides or antibodies. This strategy has been used for introducing tumor‐targeting antibody fragments on the surface of *Escherichia coli* outer membrane vesicles (OMVs) [64], as well as various heterologous antigens on the surface of Salmonella OMVs for vaccination purposes [65]. During the past decade, expansion of the Tag/Catcher toolbox beyond the SpyTag/SpyCatcher system has provided an opportunity for developing innovative methods for EVs surface modification, including SnoopTag/SnoopCatcher and DogTag/DogCatcher systems, which remain to be explored in future studies [66, 67, 68].

Protein ligation tools offer a versatile, efficient, and highly site‐specific approach for surface modification of EVs under physiological conditions, ensuring biocompatibility while maintaining EV integrity, making it an ideal approach for targeted delivery, imaging, and therapeutic applications.

### 2.4 Thiourea Bond Formation With Isothiocyanates

The electrophilic carbon of the isothiocyanate group reacts with nucleophiles in mild conditions in the absence of catalysts. In theory, they can react with thiols, amines (Figure 2F), and hydroxyl groups within biological systems, amongst which thiols show better kinetics. However, due to the reversible nature of the thiol‐isothiocyanate interaction under mild physiological conditions, the outcome ultimately shows selectivity toward amines as the final product [69]. The reaction between isothiocyanate and amine has been extensively used for polymer modification [70], but the most notable application in bioengineering is probably for labeling proteins with fluorescein isothiocyanate (FITC) [71]. Nevertheless, FITC is rarely employed for direct labeling of proteins on the EV surface. Instead, it is more commonly conjugated to antibodies targeting specific EV surface markers or immobilized on flow cytometry beads to facilitate the detection and characterization of EVs.

A major application of the isothiocyanate‐amine reaction in surface modification of EVs is to install radioisotope chelators on the surface of EVs for imaging purposes. For instance, the para‐isothiocyanate benzyl derivatives of Deferoxamine [72, 73] and 1,4,7‐triazacyclononane‐1,4,7‐triacetic acid (NOTA) [74, 75] have been extensively used for radiolabeling EVs with ^89^Zr, ^64^Cu, and ^68^Ga for pharmacokinetic studies using positron emission tomography (PET) scan methods. The same principle can be suggested for developing EV labeling strategies using novel bifunctional chelators to trap a more diverse array of radioisotopes and metal‐based contrast agents for various imaging technologies, including PET, magnetic resonance imaging (MRI), and SPECT.

Overall, because of rapid kinetics in mild physiological conditions, high selectivity for amines, and lack of problematic byproducts, the amine‐isothiocyanate reaction provides a flexible platform for conjugating dyes, metals, and radioisotopes to the surface of EVs.

## 3 Thiol‐Directed Reactions

**FIGURE 3:**
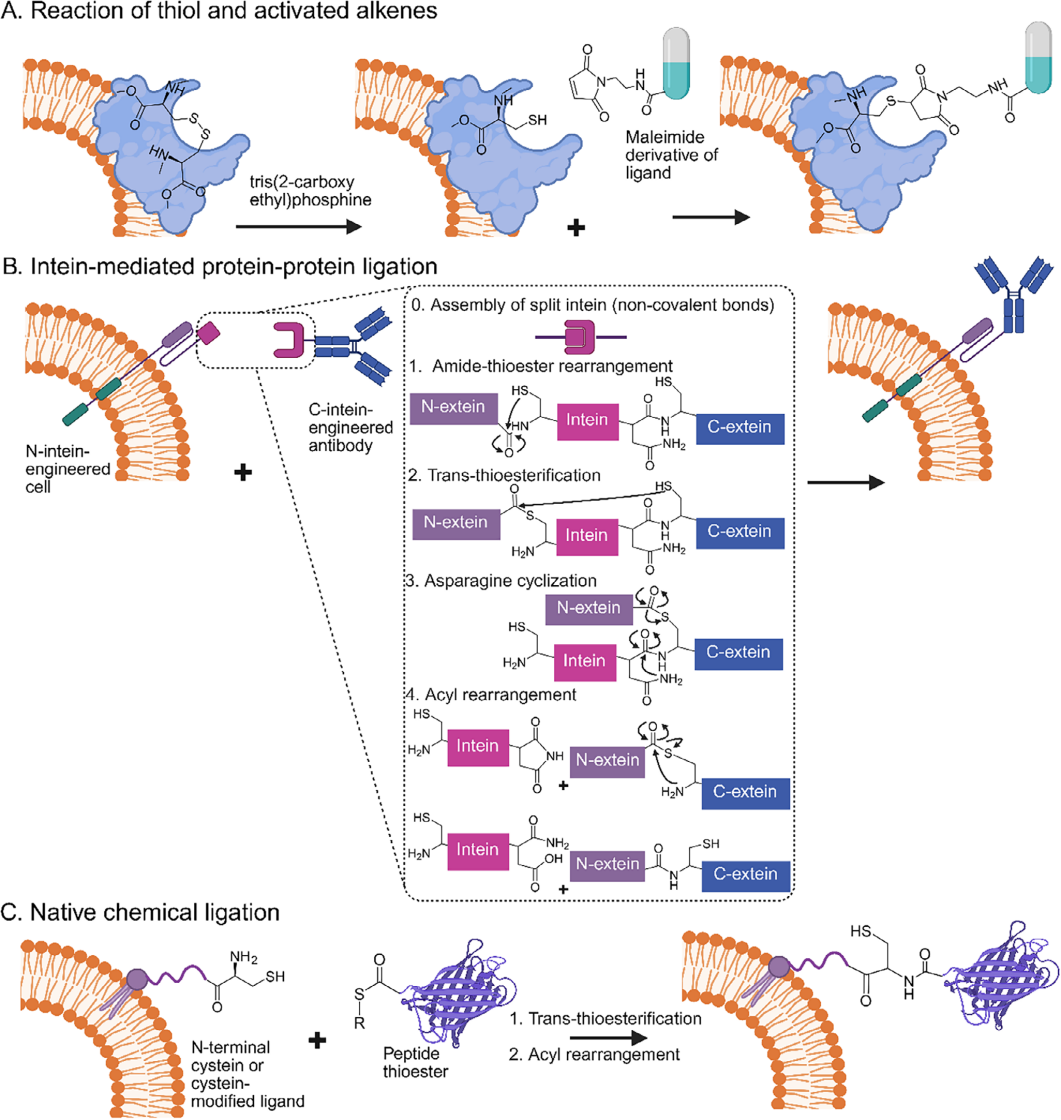
Major thiol‐directed reactions showing potential for EV surface engineering.

Thiol‐directed biocompatible reactions represent a versatile and powerful class of reactions widely employed in the engineering of biomolecules, nanoparticles, including EVs, and cells. Owing to the unique nucleophilicity and relative rarity of thiol‐bearing cysteine residues in biological systems, these reactions offer high site‐selectivity and efficiency under mild, aqueous conditions compatible with complex biological environments. A broad spectrum of thiol‐reactive strategies has been developed, including Michael‐type additions (e.g., maleimides, vinyl sulfones), alkylation reactions (e.g., haloacetamides, epoxides), disulfide exchange, and nucleophilic aromatic substitution (e.g., perfluoroaryl systems). These reactions enable covalent functionalization with a range of synthetic moieties—such as fluorophores, polyethylene glycol (PEG), targeting ligands, and therapeutic cargos—without disrupting native structures or functions. Their tunable reactivity and biocompatibility make them especially valuable in diverse applications, including targeted drug delivery, biosensing, and the development of multifunctional nanomaterials. This section explores the mechanistic principles and emerging applications of the most important thiol‐directed reactions across these various bioengineering domains, summarized in Figure 3.

### 3.1 Activated Alkenes

The Michael reaction between activated alkenes and thiols has been well documented since the 1950s [76]. Maleimides, acrylates and acrylamides, vinyl sulfones, and fumarates are examples of activated alkenes that have been used in various bioengineering avenues, including biomolecule engineering, as well as nanoparticle and hydrogel development [77, 78, 79].

The Michael addition reaction between activated alkenes and thiol proceeds via a nucleophilic attack of a thiolate anion on the electron‐deficient β‐carbon of the activated alkene, resulting in a stable thioether bond. This reaction is rapid and selective in physiological conditions [80]. However, in suboptimal pH conditions, other nucleophiles like amines compete with the thiolate ion and reduce the yield of the reaction. Besides, the stability of the thioether linkage can be influenced by the surrounding environment, with potential for retro‐Michael reactions under certain conditions, like highly basic environments [81, 82].

Thiol–maleimide chemistry has been well established in bioconjugation, particularly for linking peptides or proteins to functional moieties. Clinically relevant examples of thiol‐maleimide chemistry include FDA‐approved antibody–drug conjugates such as trastuzumab‐emtansine, in which a cytotoxic drug is covalently attached to an antibody via thiosuccinimide linkage [83]. Additional applications involve maleimide‐mediated polymer crosslinking in hydrogels [84], designing thermos‐ and redox‐responsive drug delivery systems [85, 86], cell surface modification [87, 88], and nanoparticle engineering [89, 90], including manipulation of EVs. The thiol–maleimide linkage is frequently employed in two approaches for EV surface functionalization.

In the first approach, surface‐exposed disulfide bonds on EV membrane proteins are selectively reduced with tris(2‐carboxyethyl) phosphine (TCEP) [91, 92, 93, 94, 95] and are subsequently conjugated to a maleimide‐functionalized ligand (Figure 3A), or a linker molecule containing a maleimide moiety. Examples of maleimide‐functionalized cargo encompass a variety of active molecules and nanoparticles, such as the cytotoxic agent oxaliplatin [96], radionuclide chelator 1,4,7,10‐tetraazacyclododecane‐1,4,7,10‐tetraacetic acid (DOTA) [97], the tumor targeting ligand transferrin [91], a variety of fluorescent dyes [98], and even maleimide‐DNA hinge attached to quantum dots [95].

In the second approach, maleimide is conjugated to a lipophilic anchor that spontaneously inserts into the EV membrane, followed by reacting with a functional moiety bearing free thiol residues [99, 100, 101, 102]. For instance, Di et al. demonstrated a method for extracellular vesicle (EV) enrichment using magnetic particles, leveraging the chemoselective thiol–maleimide reaction [100]. The first step involved the spontaneous insertion of maleimide‐functionalized DSPE‐PEG molecules into the EV membrane via hydrophobic interactions, followed by capturing the EVs with thiol‐functionalized magnetic particles. While this maleimide‐mediated magnetic isolation lacks the molecular specificity of antibody‐antigen approaches, it offers a rapid, scalable, and cost‐effective alternative for EV enrichment, making it suitable for applications where high‐throughput processing is prioritized.

Using bifunctional crosslinkers with NHS ester on one side and a maleimide group on the other side has also been employed for conjugating biomolecules like antibodies on the EVs surface [92]. Besides, this strategy was used for designing a controlled‐release hydrogel formulation of membrane vesicles from probiotic lactobacilli to facilitate wound healing [93]. In this study, free thiols from EVs’ surface proteins were fused to the carboxylated chitosan molecules via the bifunctional crosslinker, maleimidoacetic acid N‐hydroxysuccinimide ester (NHS‐maleimide). The controlled release provided by this formulation significantly improved the rate and molecular markers of wound healing compared to the vesicles incorporated into the same hydrogel without covalent bonding [93].

In summary, thiol–maleimide chemistry provides a versatile and efficient approach for surface modification of EVs, enabling precise attachment of targeting ligands, therapeutic agents, and diagnostic markers. Rapid reaction kinetics, compatibility with physiological conditions, and modularity make this reaction an attractive tool for enhancing EV functionality in therapeutic and diagnostic applications. Although the stability of thioether linkages under different biological conditions requires careful optimization to preserve conjugate integrity, their inherent instability can also be advantageously exploited to create environment‐responsive controlled‐release systems, enabling selective release of EVs or functional molecules in response to pH or redox changes.

### 3.2 Intein‐Mediated Protein‐Protein Ligation

Intein‐mediated ligation is a method of site‐specific protein ligation based on the natural process of protein splicing, where a protein segment (intein) excises itself from a precursor protein while joining the flanking sequence (exteins) through a peptide bond (Figure 3B) [103]. The process is usually initiated by the nucleophilic attack of an amino acid residue (usually cysteine, but also serine or threonine) at the N‐terminus of the intein on the peptide backbone. This results in the formation of a thioester (or ester) intermediate between the intein and the C‐extein. Then, the C‐terminal extein, beginning with a cysteine (also a serine or threonine), attacks the thioester, transferring the upstream extein to the C‐terminal extein through trans‐thioesterification. In this process, the intein is excised, and the upstream and downstream exteins are ligated via a native amide bond, with no residual sequence left behind. Discovered in the late 1980s in organisms like *Saccharomyces cerevisiae*, inteins soon gained recognition as versatile tools for protein engineering. Split intein technology has evolved for protein–protein ligation based on inteins divided into two parts and incorporated in two proteins, which reconstitute splicing when brought together [104].

In addition to protein engineering [103], this approach has been used for site‐specific drug delivery [105], T‐cell surface modification with chimeric antigen receptors [106], and conjugating proteins to the nanoparticle surface [107]. Although not reported for its ligation capacity, in the context of EV bioengineering, a pH‐sensitive intein with only excising function was used to release a therapeutic protein from a membrane‐incorporated EV‐sorting domain into the EV lumen [108, 109]. Evolving into functional excision/ligation strategies by the development of environment‐responsive and split inteins, intein technology presents tremendous potential for EV surface modification for various therapeutic and regenerative approaches, such as on‐demand drug release from EVs’ surface or triggered EV release from a matrix, which remain to be explored in future studies.

### 3.3 Native Chemical Ligation Between Thiols and Thioesters

First introduced by Kent and colleagues in 1994, native chemical ligation involves the reaction of a peptide bearing a C‐terminal thioester with another peptide containing an N‐terminal cysteine, resulting in a thioester‐linked intermediate that undergoes a spontaneous S‐to‐N acyl shift, forming a stable native peptide bond (Figure 3C) [110]. This approach has since expanded beyond peptide chemistry [111], becoming a cornerstone in protein semi‐synthesis, biomaterial engineering [112], and even nanoparticles’ surface manipulation [113]. Nevertheless, despite its high chemoselectivity, this strategy has not been reported for EV surface engineering because of the scarcity of N‐terminal cysteines.

### 3.4 Other Thiol‐Reactive Functional Groups

Thiol exchange is a reaction based on the nucleophile thiolate anion attacking a disulfide bond, resulting in the formation of a new disulfide bond and the release of a new thiolate. These reactions play a critical role in biological redox regulation (e.g., glutathione cycling) and have been adapted extensively for bioconjugation due to mild reaction conditions and chemoselectivity for cysteine. Particularly, thiol exchange reactions help design systems where pH or redox‐sensitive reversible covalent attachment or dynamic bond formation is desired, including antibody‐drug conjugates [114] and redox‐triggered release of nanoparticles from capture beads [115] and hydrogels [116]. A prominent example is incorporating disulfide bond‐forming amphiphilic peptides into EV membranes to block the premature release of a cytotoxic cargo. When the EVs reach their target tumor cells, due to the high glutathione content, the disulfide bonds are reduced, and the doxorubicin is released from the EVs [117].

Besides the major thiol reaction mentioned above, thiols participate in click reaction with isocyanates [118], catechol and quinone structures [119], epoxides [120], and other nucleophiles in the presence of a vinyl thianthrenium salt [121]. Although reported in bioconjugation for protein synthesis and labeling, as well as in polymer engineering for developing controlled release matrices [122], their potential for surface engineering of EVs remains to be studied in the future.

## 4 Hydroxyl‐Directed Reactions

Hydroxyl is an abundant functional group in biological environments, specifically in carbohydrates. However, selectively addressing hydroxyl groups in an aqueous system in which amines and thiols are abundant is not a trivial task.

**FIGURE 4:**
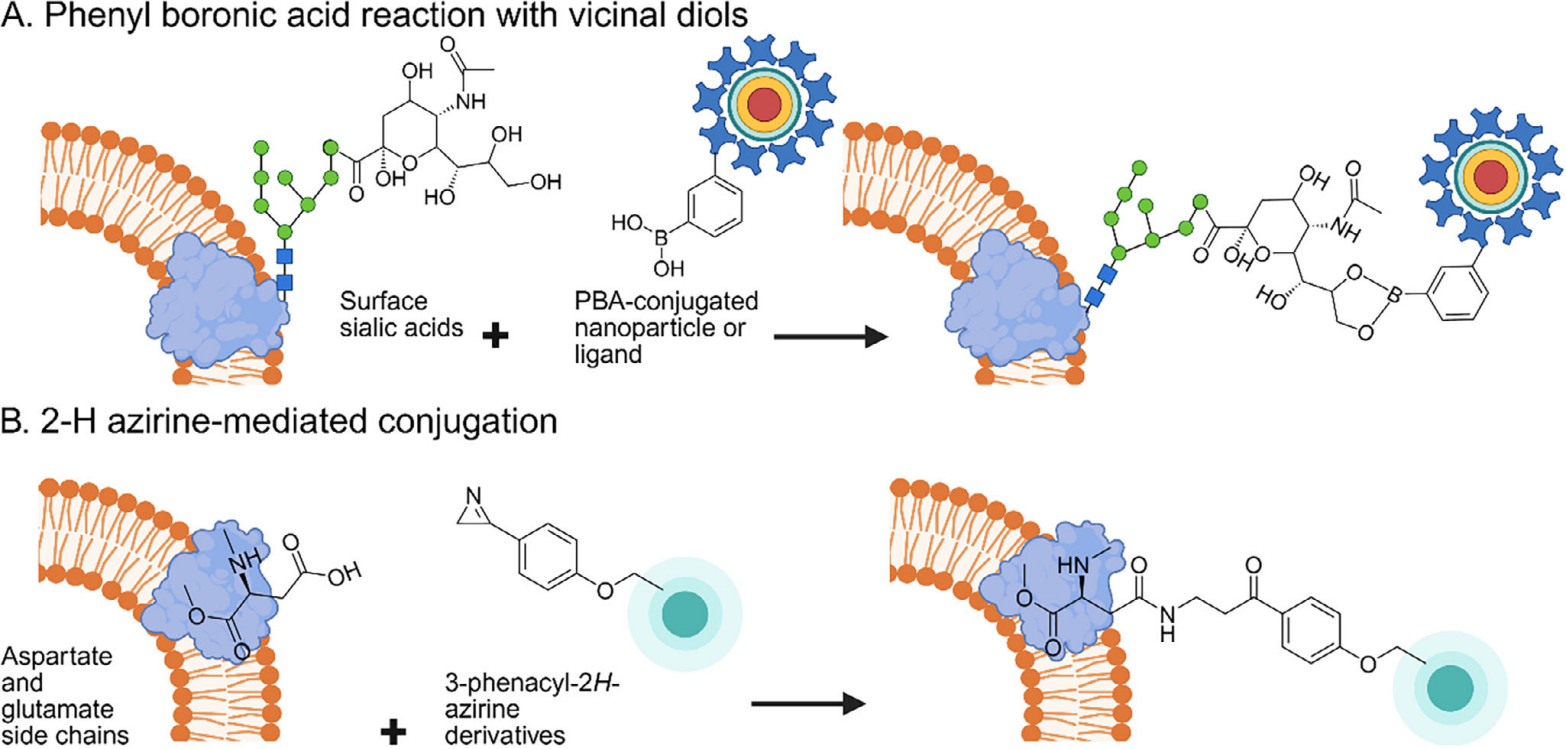
Examples of potential biocompatible (A) hydroxyl‐ and (B) carboxylic acid‐directed reactions for EV surface engineering.

Phenylboronic acid (PBA) and its derivatives are extensively used in biopolymer engineering and biosensing due to their ability to form reversible interactions with cis‐diol‐containing biomolecules, especially carbohydrates (Figure 4A) [123]. PBA functional groups can be cleaved in response to glucose or pH and therefore, have been used in environment‐responsive drug delivery systems [124]. Additionally, PBA has been utilized as a targeting moiety on cells and nanoparticles for addressing sialic acids on specific cell types [125, 126]. In the context of direct bioconjugation, Liu and colleagues introduced PBA‐decorated quantum dots for labeling cell membrane sialic acids [127]. They observed rapid internalization of nanoparticles via endocytosis after PBA‐sialic acid interactions, resulting in accumulation of the nanoparticles in the perinuclear region. Using PBA‐tagged ligands for surface modification could also be a feasible strategy for EVs due to the enrichment of sialic acids on the EV surface [12]. PBA can also be used as a strategy for entrapment and controlled release of EVs from hydrogel matrices and scaffolds designed for drug delivery and bioengineering. While PBA has not been reported for direct bioconjugation to the EVs, it has been incorporated into a bi‐functional linker for immobilization of doxorubicin on the EV surface [128]. To achieve EV surface modification, PBA groups were introduced to the EV surface proteins through EDC/NHS chemistry and further reacted with two sterically adjacent hydroxyl groups from doxorubicin. Due to the mild reaction conditions, compatibility with aqueous environments, high selectivity for glycans, and reversible conjugation, PBA derivatives diversify the portfolio of chemical reactions applicable to EV bioengineering.

## 5 Carboxylic Acid‐Directed Reactions

Addressing carboxyl groups, mainly from aspartic acid, glutamic acid, or the C‐terminal of proteins, as well as sialic acid residues of glycans, for bioconjugation is less common mainly because they have a low reactivity in physiological conditions, thus usually require activation. However, their scarcity compared to amines makes them a valuable target for site‐selective bioconjugation.

As we explained in Section 2.2., activating carboxylic acids through EDC/NHS or sulfo‐NHS pair is a common biocompatible method for bioconjugation through amide bond formation. This method is predominantly used for addressing amine groups on the EV surface by in situ activation of carboxylic acid‐containing ligands. To the best of our knowledge, using this principle for coupling amine‐containing ligands to the carboxylic acids on the EV surface has not been documented in the literature.

A novel approach for addressing carboxylic acids in physiological conditions without requiring prior activation is employing ligands with a 2H‐azirine functional group. Ma et al. developed a bifunctional group with 2H‐azirine and azide reactive sites and reported more than 96% selectivity of 2H‐azirine‐mediated conjugation for aspartic acid and glutamic acid residues in living cells (Figure 4B) [129]. In the absence of comparable carboxylic‐acid directed reactions and according to the stability of the developed azirine species in physiological conditions, its high reactivity due to the strong electrophilicity, and high stability of the ligation product—N‐phenacylacetamide—as well as rapid and site‐selective mechanism, this method remains a potential candidate to be investigated in the context of EV surface modification.

## 6 Ligand‐Directed Reactions

Ligand‐directed proximity‐induced reactions are a powerful class of biocompatible strategies for the selective covalent modification of native proteins in complex biological environments. By coupling a high‐affinity ligand with a finely tuned functional group, these approaches exploit binding‐induced proximity to accelerate covalent bond formation only at the target protein. This enables site‐specific labeling with minimal perturbation of protein function [130].

**FIGURE 5:**
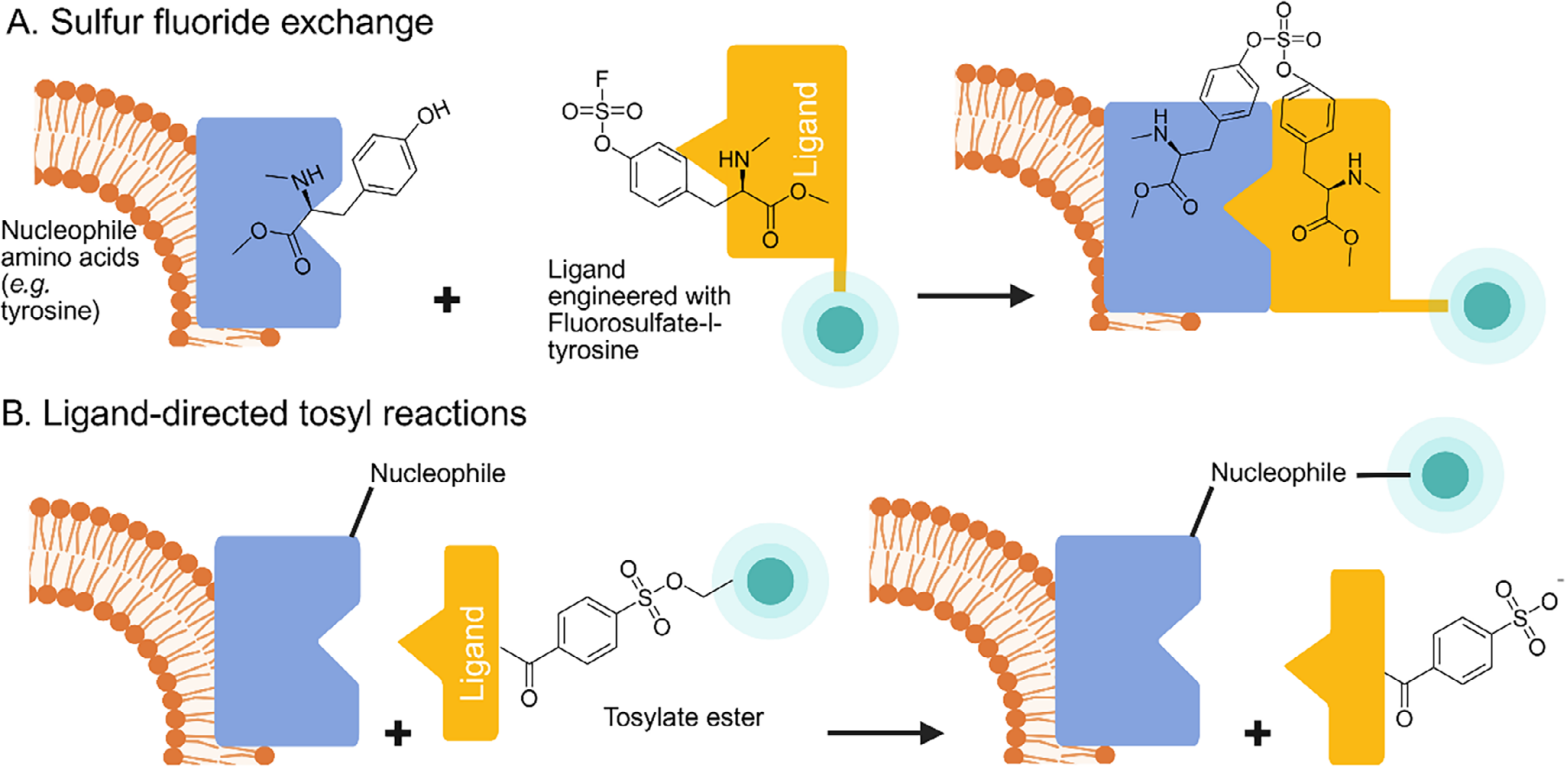
Examples of ligand‐directed reactions for engineering surface proteins, presenting potential for EV surface modification.

Sulfur fluoride exchange (SuFEx) is a powerful click chemistry platform with broad utility in protein engineering. Reintroduced by Sharpless and colleagues in 2014, SuFEx is based on the exchange of sulfur‐bound fluorine atoms in S(VI) fluorides—such as sulfonyl fluorides, fluorosulfates, and sulfamoyl fluorides—with various nucleophiles (Figure 5A) [131]. One of the most notable implementations of SuFEx involves aryl fluorosulfates, which selectively react with the phenolic hydroxyl group of tyrosine residues in proteins, enabling site‐specific labeling with minimal off‐target reactivity. This reaction is slow in bulk aqueous solution but can proceed rapidly when driven by proximity. Based on these principles, Wang et al. have genetically encoded an unnatural amino acid, fluorosulfate‐L‐tyrosine, in both bacterial and mammalian cells [132]. This amino acid underwent SuFEx with adjacent lysine, histidine, and tyrosine residues in physiological conditions, showing good biocompatibility. In another innovative approach, Serniuck and colleagues designed bifunctional small molecules—covalent immune recruiters—with an aryl fluorosulfate functional group to enhance the immune signaling upon the interaction of engineered T‐cell receptors and their target on tumor cells [133].

Ligand‐directed tosyl (LDT) reactions are another approach developed by Hamachi and colleagues for site‐selective covalent labeling of proteins [134]. The LDT reaction is a proximity‐driven nucleophilic substitution process. Accordingly, the ligand is linked to the aryl side of the tosylate, while the probe is attached to the sulfonate ester. Upon target binding, a nearby nucleophilic residue attacks the electrophilic carbon connected to the tosylate, transferring the probe to the protein and releasing the ligand. Because the ligand occupies and shields the binding pocket during labeling, the target proteins can be modified without loss of function—unlike conventional affinity labeling methods (Figure 5B) [135]. LDT has been extensively used for conjugating a variety of probes to proteins in vitro or in vivo. For instance, FKBP12 protein from T cells was labeled with a photo‐crosslinker—diazirine– to allow UV–induced protein‐protein interactions [136]. The most important advantage of this strategy compared to affinity labeling is that the active site of the target protein remains intact, retaining the protein's activity [137]. Besides, this strategy is applicable for installing a variety of functional groups and dyes on the target protein [135]. The major limitation of this approach is the availability of a small molecule or peptide‐based ligand for the target protein that can be modified with cleavable tosyl linkers.

Based on the principles of ligand‐directed reactions, diverse cleavable chemical linkers with specificity for a certain nucleophilic amino acid have been developed for labeling proteins in vitro and in vivo, amongst which, acyl imidazoles (selective for lysine, serine, and tyrosine), α‐methacrylamide (selective for cysteine), as well as N‐acyl‐N‐alkyl sulfonamides and sulfone azides (selective for lysine) can be mentioned [137]. Although none of these ligand‐directed chemistries have been used for EV surface engineering, specific surface proteins with known small‐molecule or peptide ligands are potential candidates for future studies. For instance, gefitinib with affinity for epidermal growth factor receptor (EGFR), sorafenib targeting vascular endothelial growth factor receptor (VEGFR), and various small molecules with affinity for programmed death‐ligand 1 (PD‐L1) can be used in conjunction with a cleavable linker and a probe to enable selective labeling and isolation of tumor‐specific EVs for diagnostic purposes.

## 7 Carbonyl‐Directed Reactions

Introducing electrophile carbonyls to polymers and synthetic nanoparticles is a common strategy to allow bioconjugation and controlled release through a reversible imine bond between nucleophiles (e.g., amine and thiol) and aldehydes [138, 139]. Although emerging frequently in metabolic processes, naturally occurring aldehydes and ketones are relatively scarce on the surface of living cells and EVs, limiting their direct use for bioconjugation. However, terminal sugar residues—particularly sialic acids—on glycans contain vicinal diols that can be selectively oxidized to aldehydes in a controlled condition using sodium periodate. Controlling the concentration of sodium periodate, duration, and temperature of the reaction is necessary to avoid extensive oxidative damage to other biomolecules and cell viability [140, 141]. However, aldehyde handles are uncommon for EV surface modification because they readily react with surface amines. As an alternative, metabolic engineering of cells with ketone‐containing sugars like *N*‐levulinoylmannosamine has been suggested as a robust strategy for introducing carbonyl groups to the cell surface [142]. The potential of this approach for acquiring ketone‐modified EVs remains to be investigated in the future.

### 7.1 Hydrazone Coupling

**FIGURE 6:**
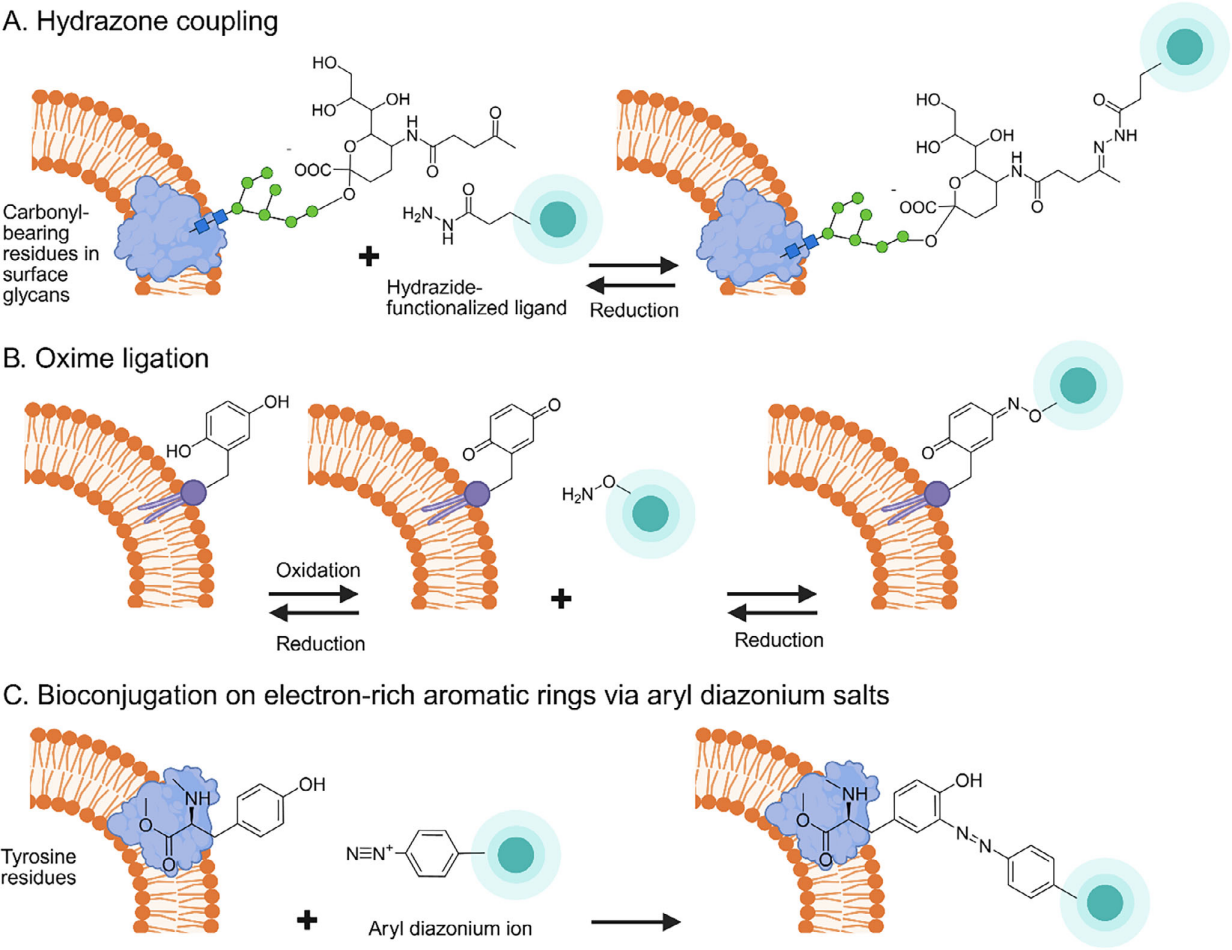
Examples of biocompatible carbonyl‐directed reactions (A and B) and bioconjugation on an electron‐rich aromatic ring, applicable to EV surface modification.

Hydrazone coupling is a biocompatible ligation strategy that enables the covalent conjugation of biomolecules through the reaction of aldehydes or ketones with hydrazide‐functionalized probes, illustrated in Figure 6A [143]. The mechanism involves nucleophilic attack of the hydrazide nitrogen on the electrophilic carbonyl carbon, leading to the formation of a hydrazone linkage following a dehydration step. This reaction is carried out under mild aqueous conditions, with optimal performance at slightly acidic to neutral pH (5.0–6.5). Catalysis with aromatic amines such as aniline can significantly enhance the reaction rates and shift the effective pH window toward neutrality, making it suitable for being employed in physiological conditions [143]. In a proof‐of‐concept study, after cell surface glycans were treated with sodium periodate, the resulting aldehydes were conjugated to hydrazide‐labeled biotin via hydrazone bond formation [140]. In an alternative approach to harness carbonyl chemistry for hydrazone coupling on the cell surface, cells can be metabolically engineered with carbonyl‐containing monosaccharides, which allows for conjugation of hydrazide‐tagged ligands [144].

Due to pH‐sensitivity, the hydrazone bond has been employed for pH‐sensitive EV and drug release from various delivery systems [145]. An example of such a strategy in the context of EV surface modification is the indirect installation of doxorubicin to the EV surface with hydrazone bonds for pH‐induced intracellular release of Doxorubicin in tumor cells’ endosomes [146, 147]. Besides, small‐molecule bifunctional linkers with a hydrazide head have been developed for the conjugation of EVs to aldehyde‐functionalized quantum dots [148] and for isolation of EVs through capture‐release platforms [149]. Utilizing hydrazone coupling for direct surface modification of EVs, especially for designing pH‐responsive delivery systems, is a potential research avenue that should be addressed in the future.

### 7.2 Oxime Ligation

Oxime ligation is a bioconjugation strategy that enables the chemoselective formation of stable oxime bonds between carbonyl groups—aldehydes or ketones—and aminooxy‐functionalized reagents under mild aqueous conditions. Mechanistically, the reaction proceeds through nucleophilic attack of the aminooxy nitrogen on the electrophilic carbonyl carbon, forming a hemiaminal intermediate that undergoes dehydration to yield the oxime bond. This reaction is efficient at physiological to mildly acidic pH (4–7), and its kinetics can be significantly enhanced in physiologic conditions by introducing aniline, making it highly compatible with live‐cell applications [143].

Oxime ligation has been used for direct cell surface engineering. Pulsipher and colleagues reported installing carbonyl groups on the cell surface through membrane fusion with liposomes [150]. The liposomes used in this study contained hydroquinone (off‐state) on the surface, which were electrochemically oxidized to quinone (on‐state), and underwent reaction with aminooxy modified ligands, nanoparticles, and cells (Figure 6B). This process can be reversed in a reductive environment, providing a platform for redox‐responsive delivery systems. Such strategies based on oxime ligation remain to be explored in the context of EV bioengineering.

While hydrazone coupling and oxime ligation are both biocompatible and proceed under mild aqueous conditions, oxime ligation generally exhibits faster kinetics and greater stability of the resulting linkage compared to hydrazones, particularly at neutral pH. Oxime ligation kinetics are also improved more significantly with anillin, making it preferable for applications requiring rapid and efficient conjugation under physiological conditions [143]. However, hydrazone coupling remains a valuable alternative, particularly when hydrazide‐functionalized reagents are more accessible or when reversible conjugation is desired.

## 8 Bioconjugation on Reactive Carbons of Aromatic Amino Acids

Targeting reactive carbon centers of aromatic amino acids offers great opportunities for site‐selective modification beyond the classical nucleophilic reactions. The electron‐rich aromatic ring of tyrosine, as well as the indole ring of tryptophan, exhibiting unique reactivity at its C2 and C3 positions, have been extensively addressed in bioconjugation studies.

Aryl diazonium salts have been optimized for site‐selective protein modification due to their reactivity toward electron‐rich aromatic systems. They engage primarily in electrophilic aromatic substitution with residues like tyrosine, forming stable azo bonds (Figure 6C). The selectivity of aryl diazonium salts toward these amino acids is dictated by various parameters, including pH of the reaction media and the activity of the diazonium species [151]. Aryl diazonium functionality has been used for PEGylation of therapeutic proteins ex vivo [152]. However, due to the high reactivity and instability in solution, in vitro and in vivo applications in cell engineering have been limited. To address this limitation, Cornejo and colleagues have suggested a redox‐responsive protecting group for a cell‐permeating diazonium derivative of a selected probe [153]. When deprotected in the reductive environment, the diazonium dye labeled non‐phosphorylated tyrosine residues in both extracellular and intracellular environments. Aryl diazonium salts have also been utilized in site‐selective protein labeling by incorporation of 5‐hydroxy tryptophan through genetic code expansion and metabolic engineering into native proteins [154]. In another approach, 4‐aminophenylalanine residues in a labeled synthetic peptide were transformed into diazonium using sodium nitrite. After the peptide was exposed to its receptor on the cell membrane, the diazonium group on the peptide reacted with the adjacent tyrosine residues, resulting in cell membrane staining [155]. The potential of these strategies for EV surface labeling remains to be investigated in the future.

## 9 Unnatural Functional Group‐Directed Reactions

### 9.1 Staudinger Ligation of Azides and Phosphines

**FIGURE 7:**
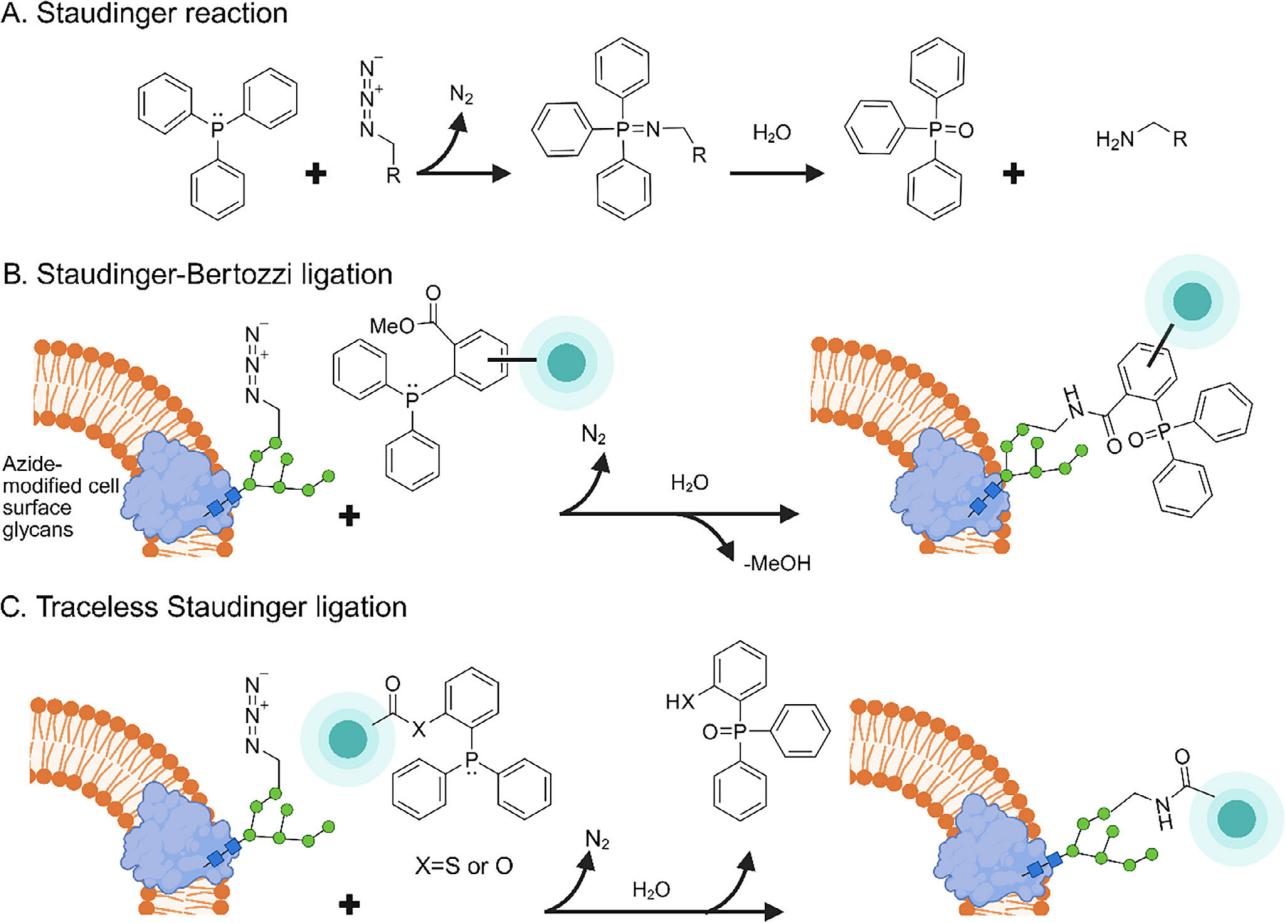
Staudinger reaction family with potential for cell and EV surface modification.

The Staudinger reaction, introduced in 1919, involves the reaction of an azide with a triarylphosphine to form an aza‐ylide (also called iminophosphorane) intermediate, which hydrolyzes into a phosphine oxide and an amine in aqueous conditions (Figure 7A) [156]. Proceeding under mild conditions without requiring a metal catalyst makes it attractive for a variety of applications. Nevertheless, the instability of the aza‐ylide in the aqueous environment suggests the need for structural optimization to make it applicable in chemical biology. The Staudinger–Bertozzi ligation modifies the original reaction by incorporating an electrophilic ester trap into the phosphine reagent, enabling the formation of stable amide linkages between azide‐functionalized biomolecules and phosphine‐labeled probes with high chemoselectivity (Figure 7B) [157]. The traceless Staudinger ligation (Figure 7C) further expands this chemistry by allowing the release of a native amide product without residual linkers, making it ideal for protein semi‐synthesis and labeling applications where minimal structural perturbation is critical.

Installation of azides on recombinant proteins through providing azide‐containing amino acids in the culture media [158] and engineering of sialic acid residues on living cells through providing azide‐containing sugars [157] paved the way for extensive application of Staudinger ligation in chemical biology. In the context of EVs and other cell‐derived nanocarriers, Staudinger‐based chemistries enable the selective and stable conjugation of targeting ligands, imaging probes, or therapeutic cargos while preserving vesicle integrity. Although employed for the installation of biomolecules and functional handles [159, 160], probes [161], and targeting moieties [162, 163] on diverse nanoparticles, the Staudinger ligation chemistry is not thoroughly investigated for EV bioengineering and has only been suggested for immobilization of azide‐modified EVs in biomaterials containing phosphine groups [164]. As interest in precision bioconjugation grows, Staudinger ligation techniques continue to be refined for enhanced kinetics, stability, and functional versatility across diverse biomedical applications, including EV bioengineering.

### 9.2 Azide‐Alkyne 1,3‐Dipolar Cycloaddition (AAC)

**FIGURE 8:**
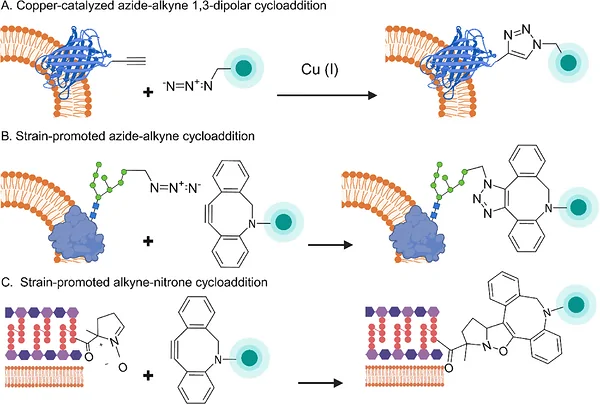
(A) Principle of copper‐catalyzed azide‐alkyne cycloaddition (CuAAC) for EV surface modification following chemical modification of available amine groups with alkyne handles. (B) Mechanism of strain‐promoted alkyne‐azide cycloaddition (SPAAC) for EV surface modification after installation of azide‐containing sugars in the surface glycans through metabolic engineering of parent cells. (C) Strain‐promoted alkyne‐nitrone cycloaddition for cell surface modification, after metabolic engineering of bacterial cells with nitrone‐containing amino acids.

The Huisgen reaction, named after the German chemist Rolf Huisgen, is the 1,3‐dipolar cycloaddition of azides with alkynes to form a mixture of 1,4‐ and 1,5‐disubstituted 1,2,3‐triazoles [165]. The ratio of 1,4‐ and 1,5‐isomers depends on steric and electronic factors but is usually unselective without a catalyst. The original form of the Huisgen reaction is typically performed in polar solvents but requires high temperatures, which makes it inapplicable to a biological context. Further, in 2002, Sharpless and Meldal introduced the paradigm‐shifting metal‐catalyzed azide‐alkyne 1,3‐dipolar cycloaddition [166, 167], giving birth to “Click Chemistry”. The introduced copper‐catalyzed azide‐alkyne 1,3‐dipolar cycloaddition (CuAAC; Figure 8A) is ∼10^7^–10^8^ times faster than the Huisgen reaction and is highly regioselective toward the 1,4‐disubstituted triazoles [17]. Furthermore, it is compatible with mild temperature and pH conditions and takes place in polar solvents, prerequisites for application in bioconjugation. In addition to copper, some other metal ions and metal complexes have been suggested as catalysts for the metal‐catalyzed azide‐alkyne 1,3‐dipolar cycloaddition, including Ruthenium, Iridium, Lanthanum, Silver, Gold, and Zinc [168]. Because of the high and unique regioselectivity, metal‐catalyzed AAC has been a powerful tool not only in bioconjugation but also in medicinal chemistry [169].

After the introduction, CuAAC soon found its way to engineering a variety of biomolecules [167] in vitro, and in viruses [170], cells [171], and even living animal models [172]. Moreover, many copper forms have been tested regarding their reactivity and biocompatibility as catalysts for the azide‐alkyne reaction. Due to the sensitivity of Cu(I) salts (chloride, bromide, iodide) to oxidation in an aqueous environment, it has been mostly employed in coordination complexes like tris(benzyltriazolylmethyl)amine (TBTA) [173] or in the form of Cu(II) salts (sulfate, chloride, acetate) together with a reducing agent (*e.g*. sodium ascorbate or TCEP (tris(2‐carboxyethyl)phosphine)) for in situ generation of Cu(I) [166]. Employing coordination complexes and reducing agents also mitigates the Cu(I)‐induced cytotoxicity by reducing the production of reactive oxygen species (ROS) and inhibiting Cu(I)’s strong interaction with specific thiol‐containing peptides and proteins [174]. Nevertheless, the possibility of cytotoxicity has limited the use of CuAAC in living systems.

The next revolutionary innovation in AAC‐based bioengineering was the development of strain‐promoted azide‐alkyne cycloaddition (SPAAC; Figure 8B) [175]. Agard et al. compared the yield of the cell surface‐installed azides’ reaction with linear and strained alkynes, as well as with Staudinger ligation reagents (a phosphine‐biotin probe) [175]. They concluded that in comparable conditions, SPAAC led to a robust surface modification, but with an efficiency lower than CuAAC and Staudinger ligation [175]. Further structural optimizations were done on strained alkynes to improve the yield of their reactions with azides, due to the excellent biocompatibility of SPAAC.

Since neither the azides nor the alkynes are present as common functional groups in biological environments, their reactions will not interfere with the parallel biochemical processes when such functional groups are introduced to the system. To harness their potential for surface modification of EVs, several strategies have been introduced based on genetic and metabolic engineering of the parent cells, as well as chemical modifications and physical interactions for installation of the azides and alkynes on the surface of EVs.

Wang et al. adapted the metabolic engineering strategy developed in Bertozzi's lab for decorating the cells with azides, in order to obtain EVs with azide‐bearing glycans [176, 177]. They co‐incubated the cells with tetraacetylated N‐azidoacetyl‐D‐mannosamine (Ac4ManNAz) or L‐azidohomoalanine (AHA) to incorporate azide‐containing units in glycans and proteins of EV‐producing cells [178]. A similar strategy based on metabolic engineering of lipids through azide choline has been developed to introduce the azide groups to the membrane lipids of EVs [179]. Such methods based on lipid‐, glyco‐, and protein engineering have been among the most well‐established strategies for introducing click handles to the surface of EVs (Figure 8B) for further reaction with a SPAAC reagent, including diagnostic dyes [180], antibodies [179], chimeric antigen receptors [181], targeting ligands [182], vaccine adjuvants [183], or EV capturing polymers with a Dibenzocyclooctene (DBCO) functional group.

Another well‐established approach for azide or alkyne installation on the EV surface is post‐isolation chemical engineering. An alkyne or azide‐containing molecule can be introduced to the native functional group through methods explained in Sections 2 and 3, amine and thiol‐directed reactions. Alternatively, our group has taken advantage of diazo transfer to primary amines, a method developed by Goddard‐Borger and Stick [184], for rapid and efficient transformation of amines to azides on the EV's surface proteins [185].

In addition to metabolic engineering and chemical modification, azide‐containing lipids have been introduced to the parent cells through incubation with azide‐containing fusogenic liposomes, leading to the presence of azides on the EV's surface [186]. Besides, azide and SPAAC handles have been introduced to the EVs’ surface through incubation with lipophilic anchors attached to the desired functional groups. For instance, it has been reported that incubation of milk‐derived EVs with cholesterol‐PEG‐DBCO provides a robust platform for surface display of azide‐modified targeting peptides [187]. The most common lipid anchor reported for installation of SPAAC handles on EVs is 1,2‐Distearoyl‐sn‐glycero‐3‐phosphorylethanolamine (DSPE), either with azide or DBCO groups [188, 189, 190, 191]. Nevertheless, dioleoyl phosphatidylethanolamine (DOPE) is suggested to outperform not only DSPE, but also ceramide and cholesterol for surface engineering of the EVs [192]. Another possibility for obtaining functionalized EVs with SPAAC handles is the hybridization of pristine EVs with functionalized liposomes. This approach is used for engineering cells [193] and liposomes [194], but not for EV modification because of being disruptive. However, using this approach for single‐step drug encapsulation and surface modification brings a significant advantage to the EV bioengineering field by reducing the required purification steps.

In conclusion, CuAAC and SPAAC offer powerful strategies for the surface engineering of EVs, with SPAAC being more favorable due to less toxicity to living systems. Azides and alkynes can be installed on the EV surface through genetic, metabolic, chemical, and physical strategies. These click chemistry approaches enable precise, stable, and efficient conjugation of functional molecules to EV surfaces without compromising their integrity or biological activity.

### 9.3 Strain‐Promoted Alkyne‐Nitrone Cycloaddition

The pioneering works of the Pezacki group expanded the bioorthogonal chemistry toolkit by introducing strain‐promoted nitrone‐alkyne cycloaddition (SPANC) [195]. In SPANC, cyclic alkynes such as DBCO undergo a 1,3‐dipolar cycloaddition with nitrones to form stable N‐alkyl isoxazolines (Figure 8C). Compared to azides, nitrones’ reaction with the strained alkynes in the same conditions follows a faster kinetic profile [195]. Besides, nitrones can be modified on both nitrogen and carbon, which provides more diversity as well as control over the reactivity of the nitrone reagents compared to azides. In addition to strained alkynes, nitrones also react with strained alkenes like trans‐cyclooctene (TCO) with a fast kinetic, diversifying the bioorthogonal labeling strategies for in vitro and in vivo reactions [196]. Although SPANC has been employed for engineering biomolecules [197, 198], cell labeling [199], and nanoparticle functionalization [200, 201, 202], using nitrones for bioengineering EVs has not yet been reported.

### 9.4 Tetrazine Ligation

**FIGURE 9:**
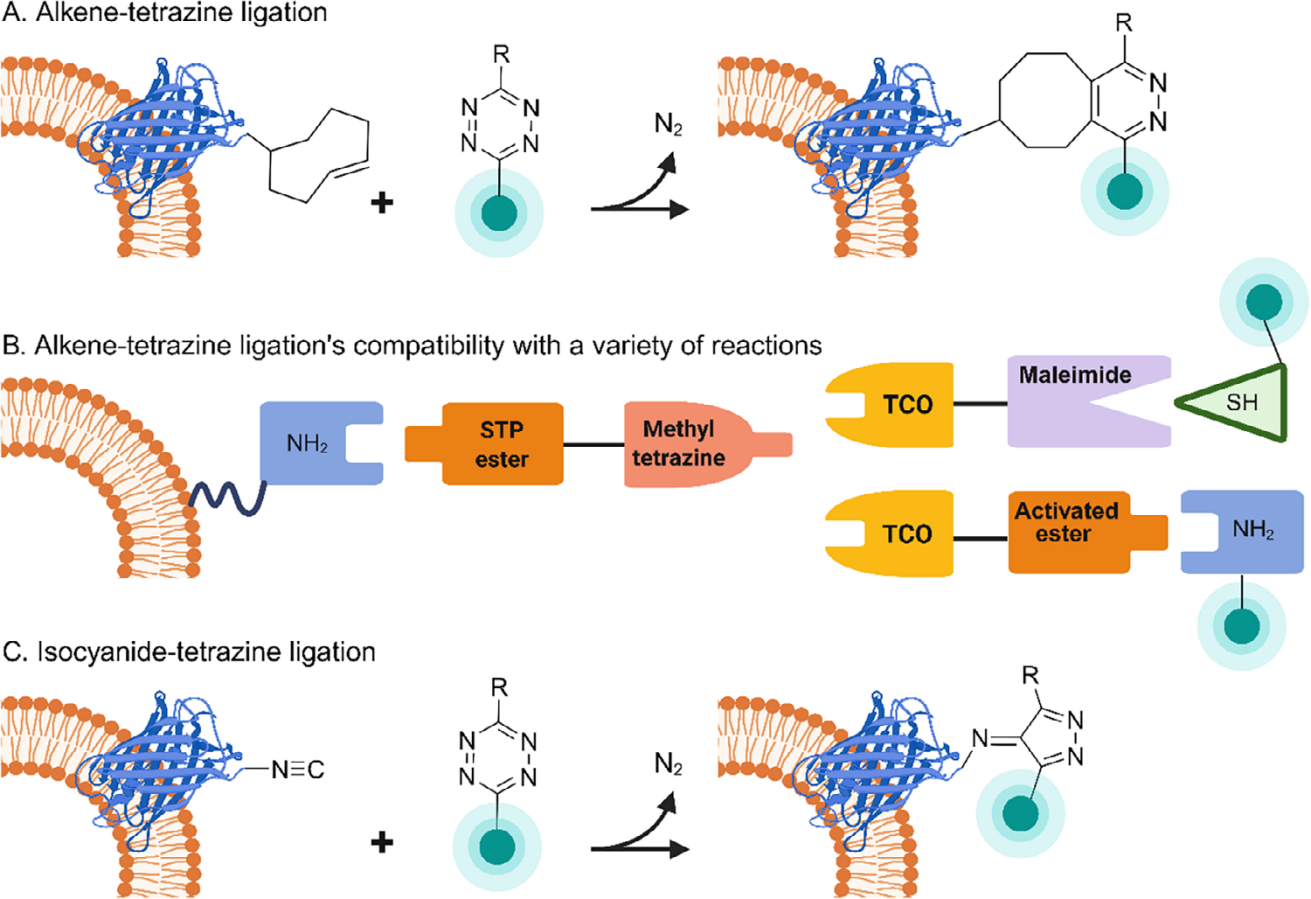
Tetrazine reactions. (A) Inverse‐electron‐demand Diels–Alder (IEDDA) reaction between 1,2,4,5‐tetrazine and trans‐cyclooctene (TCO). (B) Tetrazine ligation is orthogonal to multiple biocompatible reactions. (C) Mechanism of isocyanide‐tetrazine ligation.

The inverse‐electron‐demand Diels–Alder (IEDDA) reaction is a concerted [4+2] cycloaddition between an electron‐deficient diene, typically a 1,2,4,5‐tetrazine, and an electron‐rich or strained dienophile such as trans‐cyclooctene (TCO), norbornene, or cyclopropane (Figure 9A) [203]. It proceeds rapidly under physiological conditions, does not require a catalyst, and generates nitrogen gas as the only byproduct, minimizing toxicity. The modularity and tunability of both diene and dienophile components have also expanded IEDDA's utility in constructing and manipulating complex macromolecular architectures [204], such as nanoparticles [205], hydrogels [206], and antibody–drug conjugates [207]. Synthesis of unnatural biomolecule building blocks equipped with diverse strained alkene and alkyne groups—such as cyclopropenes [208, 209, 210], norbornenes [211], bicyclononynes [212], and trans‐cyclooctenes [212]—has enabled site‐specific integration of the IEDDA handles into nucleic acids, proteins, and extracellular matrix in living cells.

Despite the extensive use of IEDDA in vitro and in vivo for biomolecule and cell engineering, using tetrazine ligation for surface modification of EVs has been reported only in a few studies. Using antibodies or lipid anchors with a TCO or a tetrazine derivative to label the EV surface and the complementary IEDDA handle on capture beads [213, 214] or microfluidic chips [215, 216] for immobilization of EVs or isolation of a specific EV population are the most reported applications of IEDDA in EV research. Nevertheless, employing tetrazine ligation has been suggested as a modular strategy for surface engineering of EVs as well. An elaborate example of this strategy is used for conjugating antibodies or immunomodulatory ligands to the surface of erythrocyte EVs [52]. In this study, the methyltetrazine group was installed on the EVs through an amine‐reactive activated ester; then, another amine or thiol‐reactive group linked to a TCO was introduced to the system to connect the ligand on the surface of EVs (Figure 9B) [52]. Tetrazine ligation is not only compatible with thiol‐ and amine‐directed reactions, but also with other bioorthogonal approaches, including SPAAC [217, 218]. This enables designing parallel or multistep one‐pot reactions, a strategy to minimize the purification steps and increase the overall yield of the surface modification process. Such merits, as well as the exceptionally fast kinetics and biocompatibility of the IEDDA reaction, position it at the forefront of bioorthogonal chemistry.

### 9.5 Isocyanide Reactions

Isocyanide (isonitrile) is one of the smallest known bioorthogonal handles, with a potential role as both nucleophile and electrophile in diverse chemical reactions [219]. Isocyanide can be introduced to various biomolecules on the cell surface through metabolic engineering with isocyanide‐containing sugars [220], genetic code expansion technology by using isocyanide‐conjugated lysine [221], as well as through lipid tags and bifunctional chemical linkers.

Isocyanides undergo [4+1] cycloadditions with tetrazines through an IEDDA mechanism—although slower than tetrazine ligation—and form isopyrazole structures (Figure 9C). The isopyrazoles prepared from primary and secondary isocyanides are unstable in aqueous conditions, and thus have been exploited for isocyanide‐responsive activation or controlled‐release systems. Tu et al. have reported the development of isocyanopropyl‐derivatized prodrugs from cytotoxic agents and dyes, which can be activated or uncaged in the presence of tetrazine‐containing molecules or nanoparticles in vitro and in vivo [222]. On the other hand, tertiary isocyanides with higher stability in physiological conditions have been developed for bioorthogonal conjugation [223]. Stairs and colleagues metabolically modified cell membrane glycans with tertiary isocyanide derivatives of glucosamine, galactosamine, and mannosamine, which enabled cell membrane labeling with tetrazine‐conjugated dyes [220].

The isocyanide bioorthogonal chemistry has recently expanded by the introduction of isocyanide‐chlorooxime ligation. The reaction starts with the elimination of hydrochloric acid, turning the chlorooxime into reactive nitrile oxide. The intermediate then reacts with the isocyanide to generate a reactive nitrilium ion, which is subsequently captured by water to produce the final product. Bioorthogonality of this reaction was confirmed by using chlorooxime‐conjugated biotin for labeling cell surface glycans, metabolically engineered with isocyanide sugars [224].

Due to their relatively fast kinetics, bioorthogonality, and broad scope expanding from prodrug activation and controlled release to surface labeling, bioorthogonal isocyanide chemistries are potential tools to be used for EV surface modification in future research.

### 9.6 Photoclick Reactions

Photoclick chemistry is a versatile approach based on chemical reactions that are selectively triggered by light. It employs photoactivatable functional groups that remain inert under physiological conditions but can be precisely activated by light of specific wavelengths. Beyond the typical advantages of click reactions, the light‐triggered activation provides unique spatiotemporal control, enabling precise modification of biomolecules both in vitro and in vivo [225]. This combination of photo‐induction and bioorthogonality has proven especially useful for applications such as targeted bioconjugation and controlled‐release drug delivery systems.

**FIGURE 10:**
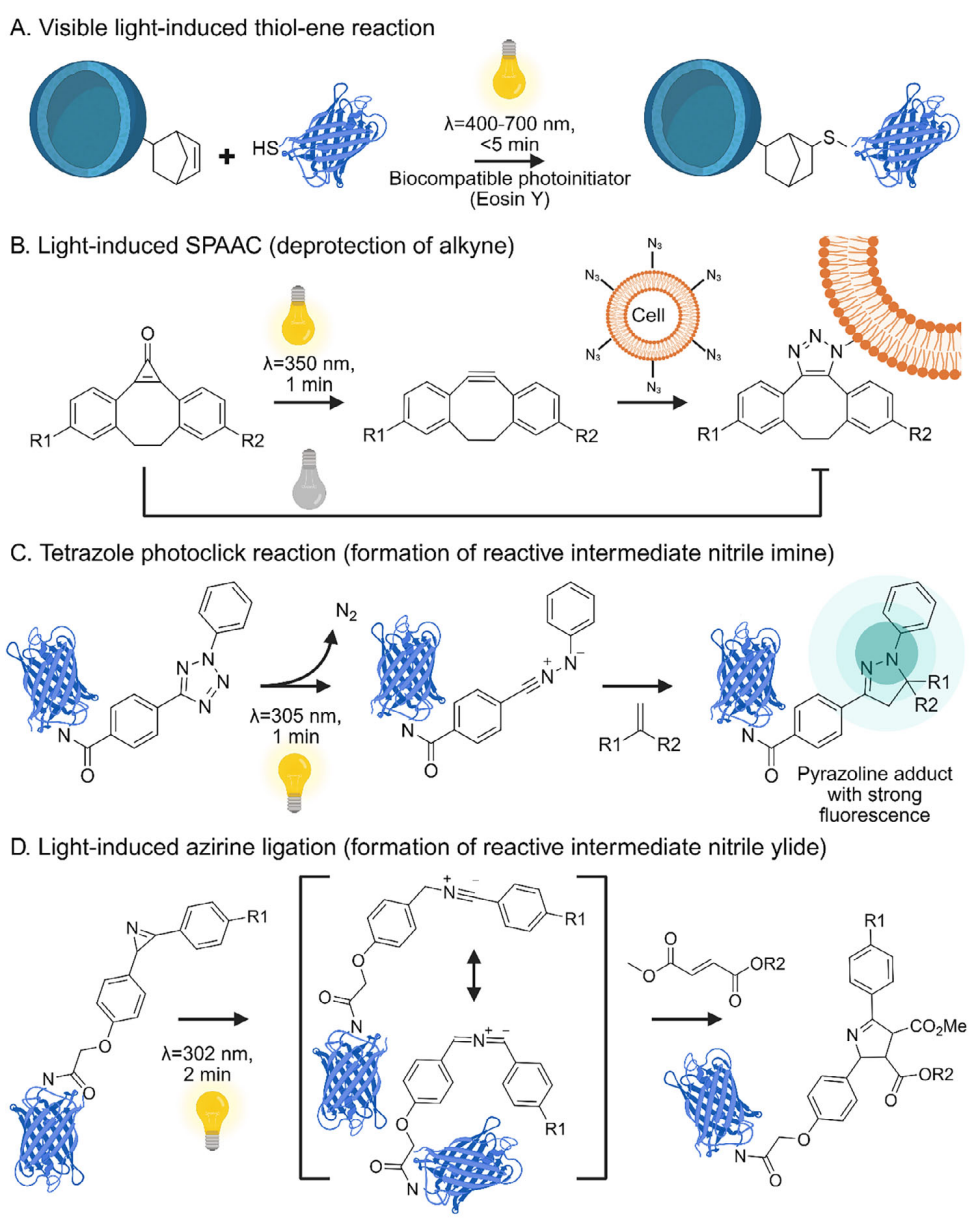
(A) Visible‐light‐induced thiol‐ene reaction. (B) Light‐induced strain‐promoted azide‐alkyne cycloaddition (SPAAC) on the cell surface. (C) Light‐induced ring opening of diaryl tetrazole followed by cycloaddition with alkenes. (D) Light‐induced ring opening of *2H*‐diarylazirine followed by cycloaddition with alkenes.

Thiol–ene and thiol–yne reactions are radical‐mediated click reactions that have emerged as robust and versatile tools in material science, nanotechnology, and bioconjugation. The thiol‐ene reaction proceeds via photochemical (or thermal) generation of a thiyl radical, which is further added across unsaturated carbon–carbon bonds to generate thioether or vinyl sulfide linkages (Figure 10A), usually with a higher regioselectivity for the less substituted carbon atom [226]. In thiol–yne reactions, a similar process occurs, but the alkyne can undergo two sequential additions, leading to bis‐thioether (dithioalkyl) products [226]. The kinetics of thiol–ene/yne reactions can be dramatically accelerated by using strained alkenes or alkynes such as norbornene and cyclooctyne, due to their ring strain that lowers the activation energy for radical addition [226, 227, 228]. This makes them particularly valuable in bioorthogonal labeling, where rapid and selective reactions under mild, physiological conditions are essential. Nevertheless, the reliance on radical generation may limit compatibility with sensitive redox‐active biomolecules, and conditions should be fine‐tuned to avoid side reactions, such as disulfide formation or oxidation of thiols prior to activation.

The light‐triggered azide‐alkyne cycloaddition has evolved from CuAAC to enable more spatial and temporal control via light activation. Initially developed to circumvent the limitations associated with the copper toxicity in biological systems, photoinduced variants employ photochemical strategies to activate either the azide or the alkyne moiety under mild, catalyst‐free conditions. These reactions proceed through the generation of reactive intermediates, such as nitrenes from aryl azides upon UV or visible light irradiation, or through the photoactivation of cyclooctyne derivatives in strain‐promoted processes [229]. Reaction conditions are generally optimized to minimize the photo‐induced damage, utilizing low‐energy UV‐A and visible or near‐infrared (NIR) light, with reaction completion achieved within minutes. An example of photo‐induced SPAAC for cell surface modification was first introduced by Popik and colleagues (Figure 10B) [230]. They designed DBCO rings, with the triple bond protected as a cyclopropenone. This cyclopropenone is not reactive to azide, but when decarbonylated under irradiation with UV light (λ = 350 nm), the unmasked triple bond on the DBCO undergoes cycloaddition with azides on the surface glycans or RNA molecules of metabolically‐engineered cells [230, 231]. Further optimization on the protected DBCO and employing NIR multiphoton activation, they achieved SPAAC with submicron resolution in tissues, suggesting improved aptitude for in vivo reactions [232].

Tetrazole photoclick reactions were introduced by Sustmann and colleagues in 1967 as another member of 1,3‐dipolar cycloaddition family [233]. Forty years later, Lin et al. reinvented this reaction in the evolving framework of click reactions for protein modification [234]. Tetrazole photoclick reaction starts with light‐triggered ring‐opening of tetrazole within a diaryltetrazole structure, releasing a nitrogen molecule and a nitrile imine dipole. The nitrile imine intermediate reacts with an electronically activated alkene and produces a fluorescent pyrazoline adduct (Figure 10C), allowing the reaction kinetics to be monitored. Initial reports used UV light (λ = 300–400 nm) to induce the reaction, but it was later shown that the reaction proceeds efficiently also through two‐photon excitation with NIR light in living cells [235]. This exceptionally rapid and precise strategy has been used for labeling bacteria and human cells and has shown excellent biocompatibility [236].

Another reaction based on photoinduced ring‐opening followed by cycloaddition is the *2H*‐diarylazirine–alkene chemistry. It involves the photochemical activation of azirines—strained three‐membered nitrogen‐containing heterocycles—under UV irradiation (λ = 302–365 nm), resulting in the formation of reactive intermediates, which react with alkenes to generate heterocyclic adducts (Figure 10D) [237]. Further development of pyrene derivatives conjugated with azirine enabled the reaction to proceed under visible light irradiation, which is more compatible with living systems [238]. This reaction is compatible with aqueous conditions, mild pH, and room to physiological temperature, and requires no metal catalysts. Besides, computational analyses have indicated that the intermediates of azirine‐ene reactions, nitrile ylides, require a lower activation energy for cycloaddition compared to the intermediates of the tetrazole‐alkene reactions, nitrile imines, which makes azirines inherently more reactive dipoles compared to tetrazoles [237].

Photoclick chemistry offers a precise and biocompatible route for engineering the EV surface, enabling rapid, catalyst‐free modification under mild conditions. Light‐triggered activation affords temporal and spatial control, allowing selective functionalization without compromising vesicle integrity or cargo. This approach facilitates the modular conjugation of targeting ligands, imaging agents, or therapeutic moieties, thereby expanding the utility of EVs in diagnostics and drug delivery. Compared to conventional bioconjugation, photoclick strategies minimize nonspecific reactions and can be adapted to complex biological environments, specifically by NIR and multiphoton stimulation approaches. Further studies are required to introduce the expanding repertoire of photoclick handles to the EVs’ surface and to integrate these handles directly into EV biogenesis pathways [239].

## 10 Radiolabeling Chemistry

**FIGURE 11:**
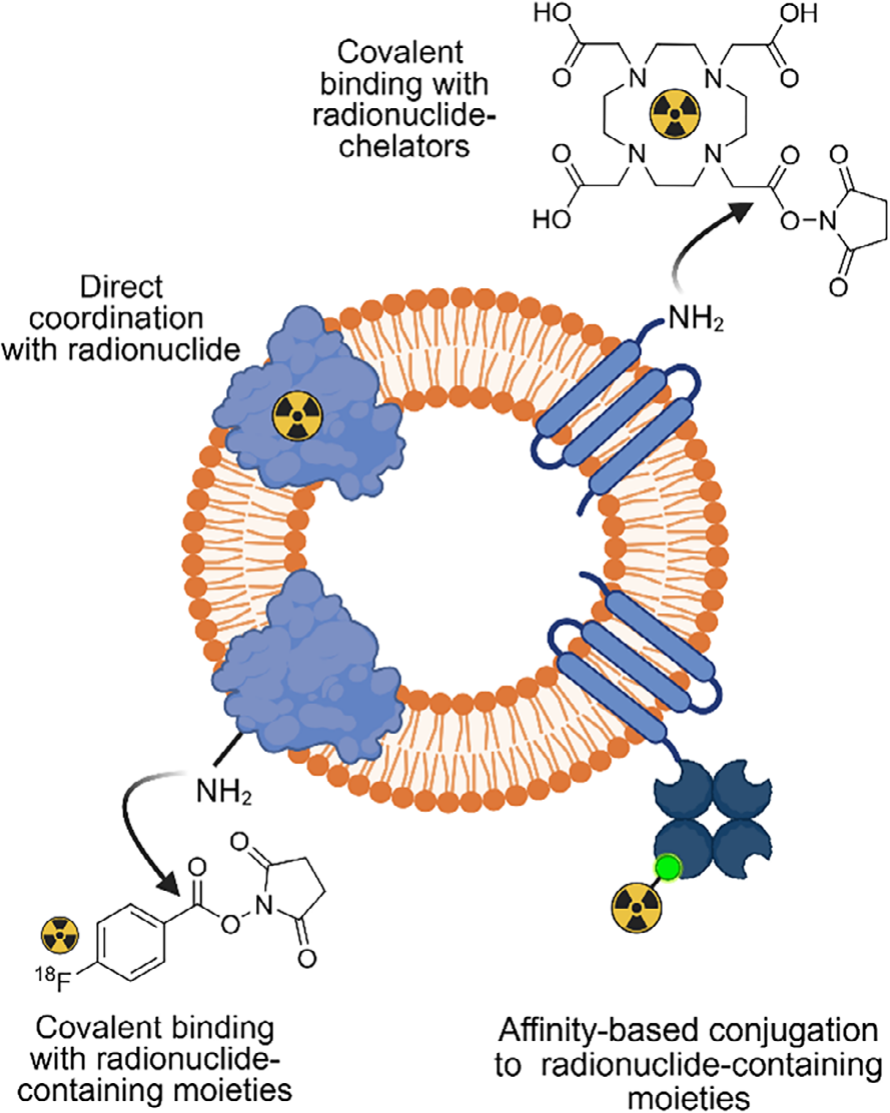
Various approaches for the installation of radionuclides on EVs’ surface.

Radiolabeling of EVs is a critical strategy for enabling biodistribution analysis and theranostic applications. The chemistry underpinning EV surface radiolabeling can be broadly classified into covalent, affinity, and coordination‐based strategies, each tailored to the physicochemical nature of the radionuclide and EV surface components. Figure 11 summarizes the strategies for radiolabeling the EVs’ surface.

Covalent radiolabeling of EVs involves the formation of stable chemical bonds between radionuclides or their prosthetic groups and functional groups on the EV surface. The most common covalent radiolabeling strategies target surface‐exposed tyrosine through electrophilic substitution (e.g., using [^1^ ^2^ ^4^I]NaI) [240] or lysine residues for amide coupling with prosthetic groups [241] such as N‐succinimidyl 4‐[^18^F]fluorobenzoate ([^18^F]SFB) [242]. This approach provides stable attachment, reducing radiolabel dissociation during circulation.

Coordination radiolabeling leverages metal‐chelator complexation for EV functionalization. Although radioisotopes can be inserted into the membrane through direct coordination interaction with natural moieties like phosphonates [243], this approach typically involves attaching a bifunctional chelator to the EV membrane—either through lipid insertion, covalent conjugation, or affinity interactions—and subsequently coordinating a radiometal ion such as ^64^Cu, ^68^Ga, ^89^Zr, ^99^Tc, or ^111^In. Common chelators include DOTA [97], NOTA [74, 75], DTPA [27], and DFO (deferoxamine) [72, 73], each selected based on the coordination preferences and kinetic inertness required for the chosen radionuclide. In addition to such bifunctional chelators, use of bioinspired coordination motifs, including catechol– [244], and histidine–metal systems [245] enable site‐specific, mild, and orthogonal EV modification.

While direct labeling through electrophilic substitution offers simplicity and may be suitable for rapid, short‐term studies, indirect chelator‐based labeling provides a more robust, flexible, and clinically relevant approach, particularly for applications requiring high in vivo stability, imaging fidelity, and regulatory scalability. Direct electrophilic substitution is largely limited to radioactive halogens (^18^F, ^123^I, ^124^I, ^131^I), which restricts its use to a narrower subset of applications and imaging time windows, while chelator‐based labeling accommodates a wide range of metallic radionuclides, expanding the choice of imaging modalities (PET/SPECT) and half‐life matching for different pharmacokinetics. Besides, Electrophilic substitution, while chemically simpler, often requires oxidizing agents that might damage the EVs and make the clinical translation more complicated.

## 11 Perspective

The advancement of EV surface engineering can benefit greatly from lessons learned in nanoparticle, cell surface, and protein engineering fields. Nanomedicine has established how surface charge, hydrophilicity, and protein corona formation influence biodistribution, circulation, and immune recognition, providing important design principles for improving EV stability and targeting. Cell surface engineering contributes strategies for selective membrane functionalization, bioorthogonal conjugation, and modulation of cell–cell interactions, which can be adapted to preserve EV membrane integrity while enhancing delivery efficiency. Meanwhile, protein engineering offers approaches to genetically or chemically modify EV membrane proteins with targeting ligands, stimuli‐responsive domains, or imaging functionalities while maintaining biological activity. Together, these fields provide complementary tools for controlled functionalization, improved reproducibility, and multifunctional design, helping transform EVs into more programmable, stable, and clinically translatable therapeutic delivery systems.

The chemical surface modification of EVs has entered a phase of rapid methodological diversification, driven by the demand for precision‐targeted therapeutics, enhanced circulation stability, and controlled bioactivity. While biological engineering of EV‐producing cells remains a robust approach, post‐isolation chemical modification offers a level of orthogonality and tunability unmatched by genetic methods, specifically for nonbiogenic chemical moieties such as small‐molecule drugs and targeting moieties. Table 1 summarizes the chemical strategies in the context of EV surface engineering, the extent of their use, and their potential applications beyond the current state of the art.

**TABLE 1:** Current extent of application of the chemical engineering avenues and their (potential) applications in developing precision therapeutics.

Approach	Reaction	Current extent of application in EV research	(Potential) Applications in precision therapeutics
Amine‐directed reactions	Schiff base reaction with aldehydes	Limited due to reversible bond formation	Controlled release of EVs from dosage form On‐demand release of cargo from EV surface
	Amide coupling with carboxylic acids, esters, and their activated species	Frequent application of NHS esters Limited exploration of other activated esters for better stability, improved kinetics, and restriction of bioconjugation to the surface Limited reports on application of anhydrides	Robust bioconjugation of various targeting ligands, dyes, PEG, and bifunctional linkers Avoiding the penetration of reagents to the EV lumen by optimization of ester reagents Simultaneous bioconjugation and surface charge‐tuning with anhydrides
	Protein‐protein ligation through amine‐directed reactions	Very limited due to the novelty of available toolbox	Highly site‐specific surface modification Expanding with the discovery of novel enzymatic tools and superglue systems
	Thiourea bond formation with isothiocyanates	Limited due to possible reactivity with thiols in specific conditions	Established bioconjugation strategy to metal and radioisotope chelators
Thiol‐directed reactions	Activated alkenes	Frequent application of thiol‐maleimide reaction	Robust bioconjugation of various targeting ligands, dyes, PEG, and bifunctional linkers pH or redox‐responsive release of EVs from matrices Stimuli‐responsive release of active compound from EVs’ surface
	Intein‐mediated protein‐protein ligation	Limited due to process complexity	Site‐selective bioconjugation Stimuli‐responsive release of EVs from matrices Stimuli‐responsive release of active compound from EVs’ surface Expanding with the development of novel biotechnological tools like split‐inteins
	Native chemical ligation between thiols and thioesters	Unexplored in the context of EVs	Site‐selective bioconjugation
	Thiol exchange	Limited due to reversible bond formation	Site‐selective bioconjugation pH or redox‐responsive release of EVs from matrices Stimuli‐responsive release of active compound from EVs’ surface
	Reactions with isocyanate, catechol, and quinone structures, and epoxides	Unexplored in the context of EVs	Site‐selective bioconjugation pH or redox‐responsive release of EVs from matrices Stimuli‐responsive release of active compound from EVs’ surface
Hydroxyl‐directed reactions	Reaction with phenylboronic acid derivatives	Limited direct application due to competing nucleophiles with hydroxyl groups and reversible bond formation	Indirect bioconjugation Stimuli‐responsive release of EVs from matrices Stimuli‐responsive release of active compound from EVs’ surface
Carboxylic acid‐directed reactions	Reaction with 2H‐azirine	Unexplored in the context of EVs	Site‐selective bioconjugation
Ligand‐directed reactions	Sulfur fluoride exchange Ligand‐directed tosyl reactions	Unexplored in the context of EVs	Selective labeling and affinity‐based isolation of EVs for diagnostic purposes Expanding toolbox with synthesizing small‐molecule ligands for specific surface proteins
Carbonyl‐directed reactions	Hydrazone coupling	Limited application due to slow kinetics in pH‐neutral conditions and pH‐sensitivity of the formed bond	Stimuli‐responsive release of EVs from matrices Stimuli‐responsive release of active compound from EVs’ surface
	Oxime ligation	Unexplored in the context of EVs	Stimuli‐responsive release of EVs from matrices Stimuli‐responsive release of active compound from EVs’ surface
Bioconjugation at an electron‐rich aromatic ring	Reaction with aryl diazonium salts	Unexplored in the context of EVs	Site‐selective bioconjugation Possibility of stimuli‐responsive activation of protected reagents
Unnatural functional group‐directed reactions	Staudinger ligation of azides and phosphines	Limited application due to availability of more novel bioorthogonal approaches	Robust bioconjugation of various targeting ligands, dyes, PEG, and bifunctional linkers
	Azide‐alkyne 1,3‐dipolar cycloaddition	Limited application of metal‐catalyzed version (CuAAC) due to biocompatibility concerns Frequent application of catalyst‐free reaction (SPAAC)	Robust bioconjugation of various targeting ligands, dyes, PEG, and bifunctional linkers
	Strain‐promoted alkyne‐nitrone cycloaddition	Unexplored in the context of EVs	Robust bioconjugation of various targeting ligands, dyes, PEG, and bifunctional linkers
	Tetrazine ligation	Limited application due to novelty and	Robust bioconjugation of various targeting ligands, dyes, PEG, and bifunctional linkers
	Tetrazine‐isocyanide reactions	Unexplored in the context of EVs	Robust bioconjugation of various targeting ligands, dyes, PEG, and bifunctional linkers Possibility of stimuli‐responsive activation of protected reagents
	Photoclick reactions	Unexplored in the context of EVs	Site‐selective bioconjugation Possibility of stimuli‐responsive activation of protected reagents Robust bioconjugation of various targeting ligands, dyes, PEG, and bifunctional linkers Expanding with the development of novel light‐controlled chemical tools
Radiolabeling chemistry	Coordination with radionuclides Affinity conjugation Covalent binding	Limited direct application Frequent application through antibody‐ligand and streptavidin‐biotin pairs Frequent application, especially with radionuclide chelators	Robust labeling based on application and specific radionuclide

Besides method‐specific explorations, several challenges must be addressed in future studies to pave the way for clinical translation of chemically engineered EVs [246]. Table 2 provides an overview of the major research directions for future efforts.

**TABLE 2:** Translational gaps and research priorities to bridge them.

Translational gaps	Research directions
Optimization of surface engineering strategies	Comparison of specificity, yield, and bioorthogonality of available chemistries Compatibility of surface engineering with cargo engineering methods
Biological consequences of surface modification	Changes in conformation and activity of modified biomolecules on EVs’ surface Changes in particle corona, biodistribution, clearance, cellular uptake, and endosomal escape
Stability and formulation considerations	Stability of modified EVs Effect of modification strategy on stability of EVs Developing optimal preservation methods for chemically engineered EVs Developing dosage forms compatible with chemically engineered EVs
Manufacturing robustness	Standardizing and scaling upstream procedures such as cell source and cultivation protocols Standardizing and scaling downstream procedures such as isolation, purification, and chemical manipulation Adaptation of advanced technologies such as microfluidics for real‐time in‐process quality corol
Regulatory framework	Development of reference materials for analysis Defining measurable parameters for deviation from standard references Establishing the purity parameters and range of impurities Establishing quantifiable functional parameters like the biosimilarity concept for biomolecular therapeutics

First, most of the available research data is at the proof‐of‐concept level for a specific chemical approach or a specific type of vesicle intended for a single purpose, without optimization of the reaction parameters or claiming universality. Systematic head‐to‐head comparisons of multiple chemistries across multiple types of EVs and multiple applications are among the most important research gaps to be addressed in future.

Second, addressing manufacturing consistency is vital. EVs are inherently heterogeneous—subject to variability in production conditions, isolation protocols, and source cell states—even when using the same chemical conjugation strategies. Preparing EV‐inspired vesicles based on hybridization of EVs and other materials, such as liposomes, can address the heterogeneity problem and improve the consistency in physicochemical characteristics. Overall, achieving batch‐to‐batch reproducibility will require rigorous standardization of workflows, adoption of reference materials, and real‐time in‐process quality control (e.g. monitoring conjugation density, surface marker display, and retained functionality) with novel chip‐based technologies, particularly during scaling [247].

Third, purification technologies must evolve beyond the traditional ultrafiltration, ultracentrifugation, and precipitation, since these methods often cannot fully separate engineered EVs from free reagents, aggregates, and non‐engineered EVs. Advanced methods—such as integrated microfluidics or affinity‐based purification—should be adapted for downstream workflows that accompany chemical modifications, ensuring minimal contamination and preserving functional moieties.

Fourth, the interplay between surface chemistry and EV biodistribution, uptake, endosomal escape mechanisms, and immunogenicity requires systematic exploration because subtle modifications may have disproportionate effects on the in vivo fate of engineered EVs.

Fifth, long‐term storage stability of functionalized EVs remains underexplored. Surface modification can accelerate degradation or aggregation over time. Studies optimizing lyophilization, cryopreservation, or buffer formulations tailored for chemically modified EVs will be essential for ensuring storage stability and clinical readiness.

Sixth, beyond lab‐scale feasibility, clinical translation demands workflows compatible with GMP, scalability, and regulatory acceptance. Establishing well‐defined specifications for acceptable modification ranges (such as percentage of modified amines), impurity thresholds (such as amount of apolipoproteins and free chemical reactants), and functional consistency (such as MIC, IC_50_, and EC_50_) will accelerate clinical translation.

In conclusion, the chemical toolbox for EV surface engineering is rich and expanding. Simultaneous or sequential bioorthogonal reactions have enabled creating EVs with multiplexed surface functionalities such as targeting ligands, stealth polymers, and imaging tags. Yet, unraveling the true potential of chemically‐modified EVs will depend on resolving manufacturing, characterization, stability, and regulatory translational challenges. By aligning reaction innovation with systemic production strategies, multidisciplinary analytics, and green, scalable protocols, the field can progress toward clinical realization of EVs as versatile nanotherapeutics and diagnostics.

## Funding

This work received funding from the European Research Council (ERC) under the European Union's Horizon 2020 research and innovation program (Grant Agreement Nos. 945602, Gels4Bac) and DATIPilot‐Sprint–NAVET (03DPS1138A) from the German Federal Ministry of Research, Technology, and Space.

## Conflicts of Interest

The authors declare no conflicts of interest.

## Data Availability

The authors have nothing to report.
